# Transplantation of Allogeneic Pericytes Improves Myocardial Vascularization and Reduces Interstitial Fibrosis in a Swine Model of Reperfused Acute Myocardial Infarction

**DOI:** 10.1161/JAHA.117.006727

**Published:** 2018-01-22

**Authors:** Valeria Vincenza Alvino, Rodrigo Fernández‐Jiménez, Iker Rodriguez‐Arabaolaza, Sadie Slater, Giuseppe Mangialardi, Elisa Avolio, Helen Spencer, Lucy Culliford, Sakinah Hassan, Lorena Sueiro Ballesteros, Andrew Herman, Ali Ayaon‐Albarrán, Carlos Galán‐Arriola, Javier Sánchez‐González, Helena Hennessey, Catherine Delmege, Raimondo Ascione, Costanza Emanueli, Gianni Davide Angelini, Borja Ibanez, Paolo Madeddu

**Affiliations:** ^1^ Bristol Heart Institute School of Clinical Sciences University of Bristol United Kingdom; ^2^ School of Cellular and Molecular Medicine University of Bristol United Kingdom; ^3^ Centro Nacional de Investigaciones Cardiovasculares Carlos III (CNIC) Madrid Spain; ^4^ The Zena and Michael A. Wiener Cardiovascular Institute Icahn School of Medicine at Mount Sinai New York NY; ^5^ Adult Cardiac Surgery Department La Paz University Hospital Madrid Spain; ^6^ Philips Healthcare Madrid Spain; ^7^ Bristol Genetics Laboratory Southmead Hospital Bristol United Kingdom; ^8^ IIS‐Fundación Jiménez Díaz Hospital Madrid Spain; ^9^ Centro de Investigación Biomédica en Red Cardiovascular (CIBERCV) Madrid Spain

**Keywords:** angiogenesis, cell therapy, large animal models, myocardial infarction, pericytes, Angiogenesis, Cell Therapy, Vascular Biology

## Abstract

**Background:**

Transplantation of adventitial pericytes (APCs) promotes cardiac repair in murine models of myocardial infarction. The aim of present study was to confirm the benefit of APC therapy in a large animal model.

**Methods and Results:**

We performed a blind, randomized, placebo‐controlled APC therapy trial in a swine model of reperfused myocardial infarction. A first study used human APCs (hAPCs) from patients undergoing coronary artery bypass graft surgery. A second study used allogeneic swine APCs (sAPCs). Primary end points were (1) ejection fraction as assessed by cardiac magnetic resonance imaging and (2) myocardial vascularization and fibrosis as determined by immunohistochemistry. Transplantation of hAPCs reduced fibrosis but failed to improve the other efficacy end points. Incompatibility of the xenogeneic model was suggested by the occurrence of a cytotoxic response following in vitro challenge of hAPCs with swine spleen lymphocytes and the failure to retrieve hAPCs in transplanted hearts. We next considered sAPCs as an alternative. Flow cytometry, immunocytochemistry, and functional/cytotoxic assays indicate that sAPCs are a surrogate of hAPCs. Transplantation of allogeneic sAPCs benefited capillary density and fibrosis but did not improve cardiac magnetic resonance imaging indices of contractility. Transplanted cells were detected in the border zone.

**Conclusions:**

Immunologic barriers limit the applicability of a xenogeneic swine model to assess hAPC efficacy. On the other hand, we newly show that transplantation of allogeneic sAPCs is feasible, safe, and immunologically acceptable. The approach induces proangiogenic and antifibrotic benefits, though these effects were not enough to result in functional improvements.

**Clinical Perspective**

**What Is New?**

- Pericytes are a promising cell product for cardiovascular repair but experimental evidence of safety and efficacy has been obtained in murine models only.
- This is the first cell therapy study of human and swine pericytes in a large animal model of reperfused myocardial infarction.
- Results show safety of the cell product. Pericyte transplantation induces proangiogenic and antifibrotic benefits, though these effects were not enough to result in functional improvements as assessed by magnetic resonance imaging.

**What Are the Clinical Implications?**

- Triangulation of present data and previous studies in mice suggest that pericyte therapy might be especially amenable to patients with nonrevascularizable coronary artery disease unresponsive to optimal treatment.
- Since pericytes improve angiogenesis and inhibit fibrosis, conjoint cardiomyogenic treatments may be necessary to achieve full restoration of both perfusion and contractile function.

## Introduction

Coronary artery disease is the leading cause of death worldwide, accounting for over 17 million deaths annually. Ischemic heart failure has reached epidemic proportions, accounting for an estimated 26 million people worldwide and resulting in more than 1 million hospital episodes annually in the United States and Europe.^1^

Transplantation of proangiogenic cells following an acute myocardial infarction (MI) may help prevent the infarct extension by promoting cardiomyocyte rescue, awakening the hibernated myocardium in the area at risk, and modulating inflammation and fibrosis.^2^, ^3^ Cell therapy of coronary artery disease has evolved rapidly from bench to bedside; however, despite positive results in murine models of MI, small‐ or medium‐sized clinical studies and meta‐analyses have only shown modest benefit in humans.^4^, ^5^, ^6^, ^7^ This could be principally because of the use of inappropriate and ill‐defined cell populations, which calls for a continued search for optimal, quality‐assured cell products. Moreover, very few studies have adopted the robust approach to forecasting clinical efficacy through validation in both small and large animal MI models before moving to humans.^8^

Different mesenchymal cell populations have been isolated from the adventitial layer of murine and human vessels.^9^, ^10^, ^11^, ^12^, ^13^, ^14^, ^15^ In a seminal study, we discovered the presence of CD133^+^/CD34^+^/KDR^+^ cells endowed with potent vasculogenic activity in the human fetal aorta.^14^ Searching for a similar angiogenic cell population in the human adult vasculature, we have focused on adventitial pericytes (APCs) that coexpress in situ the classical markers NG2 and PDGFRβ and the cell surface glycoprotein CD34. Using a Good Manufacturing Practice–compliant standard operating protocol (SOP), which consists of an initial immunosorting for the CD34 antigen and exclusion of CD31^+^ cells followed by adhesive culture/expansion in a selective culture media, we were able to expand millions of viable human APCs (hAPCs) from a small remnant of the saphenous vein used for coronary artery bypass graft surgery.

Expanded hAPCs share properties with mesenchymal stem cells, including the expression of CD44, CD90, CD105, and CD73, the lack of hematopoietic (CD45) and endothelial markers (CD31, von Willebrand factor, and Vascular Endothelial‐cadherin), as well as clonogenicity, and immunomodulatory activity. In a series of studies conducted in mice, we demonstrated that cell therapy with hAPCs has a remarkable therapeutic potential for the treatment of peripheral and myocardial ischemia.^9^, ^16^, ^17^, ^18^ In particular, transplantation of hAPCs promotes cardiac repair in immunodeficient and immunocompetent murine models of MI, through incorporation into the host's vasculature and the release of angiocrine and antifibrotic factors.^9^, ^16^, ^17^ Haplotype analysis and cytotoxicity assays confirmed tolerance of hAPCs by the mouse immune system.^16^ We have also conducted comparative studies in mice with MI, where hAPCs were shown to be superior to cKit+ cardiac stem cells in terms of proangiogenic activity. They also surpassed bone marrow–derived mesenchymal stromal cells in improving contractility indices and not causing calcification, which was frequently observed in mesenchymal stromal cells–transplanted hearts.^9^, ^16^

In the attempt to make a further step towards clinical translation, here we report the findings of the first‐ever large animal trial of xenogeneic and allogeneic APC therapy in a swine model of reperfused MI.

## Materials and Methods

An extended version with details of experimental procedures is provided in Data S1. The data, analytic methods, and study materials will be made available upon request to other researchers for purposes of reproducing the results or replicating the procedure.

### Ethics

Institutional Review Board approval was obtained according to the guidelines noted in the Instructions to Authors. The collection of long saphenous vein leftovers from coronary artery bypass graft surgery complied with the principles stated in the Declaration of Helsinki and was approved by the Bath Research Ethics Committee (REC reference 06/Q2001/197). Patients gave written informed consent for inclusion in the study. Available clinical data of vein donors together with specific use of corresponding hAPC lines are shown in Table 1.

**Table 1:** Clinical Characteristics of Human Donors and Specific Use of Corresponding hAPC Lines

Patient Code	Age, y	Sex	Risk Factors/Pathology/Intervention									hAPC Line/Use	
			Smoker	DM	MI	HPT	HPC	CPD	CHF	AF	PCI	Line	Study
040613 B	72	M	No	No	No	Yes	No	No	No	No	No	1	Efficacy
061213 D	78	F	No	No	No	Yes	Yes	Yes	Yes	No	No	2	Efficacy
180314 D	78	M	Ex	No	Yes	Yes	Yes	No	No	Yes	No	3	Efficacy
140414 A	66	F	No	No	Yes	Yes	Yes	No	No	No	No	4	Cytotoxic assay
300414 F	57	M	Yes	No	Yes	Yes	Yes	No	No	No	No	5	Efficacy
070514 A	72	M	No	No	Yes	Yes	No	No	No	No	No	6	Efficacy
080514 C	71	M	Ex	No	No	Yes	Yes	No	No	No	Yes	7	Efficacy
080514 D	84	M	Yes	No	Yes	Yes	Yes	Yes	No	No	No	8	Efficacy
030614 E	66	M	Yes	No	Yes	No	Yes	No	No	No	No	9	Cytotoxic assay
140115 B	66	M	Ex	Yes	Yes	Yes	Yes	No	No	Yes	No	10	Engraftment
160115 A	54	F	Yes	No	Yes	Yes	No	No	No	No	Yes	11	Engraftment
090215 A	72	M	Ex	No	No	Yes	No	No	No	Yes	No	12	Engraftment
200215 B	67	F	Yes	No	Yes	Yes	Yes	No	No	No	Yes	13	Engraftment
171115 A	67	M	Yes	No	Yes	No	Yes	No	No	No	No	14	Karyotyping
171115 B	68	M	No	No	Yes	Yes	Yes	No	No	No	No	15	Karyotyping
120116 A	80	M	Ex	Yes	No	Yes	Yes	No	No	No	Yes	16	Karyotyping
120116 B	71	M	No	Yes	Yes	Yes	Yes	No	Yes	Yes	No	17	Karyotyping
090216 B	73	M	Ex	No	Yes	Yes	Yes	No	No	No	No	18	Karyotyping

APCs were isolated from saphenous vein leftovers of patients undergoing CABG surgery. Expanded hAPC lines (identified with a sequential numerical code from 1 to 18) were used for the following studies: (1) Intramyocardial transplantation in swine with MI to assess feasibility/efficacy of cell therapy; (2) Intramyocardial transplantation in swine with MI to assess engraftment; (3) Assessment of genetic stability following in vitro expansion; and (4) Assessment of immunogenic activity. AF indicates atrial fibrillation; CABG, coronary artery bypass graft; CHF, congestive heart failure; CPD, chronic pulmonary disease; DM, diabetes mellitus; Ex, Ex‐smoker (patient who stopped smoking at least 1 month before the surgery); hAPC, human adventitial pericytes; HPC, hypercholesterolemia; HPT, hypertension; MI, myocardial infarction; PCI, percutaneous coronary intervention.

The in vivo studies on swine were conducted in accordance with institutional guidelines and the Guide for the Care and Use of Laboratory Animals (the Institute of Laboratory Animal Resources, 1996). In particular, experimental protocols received the approval of the UK Home Office (licences 30/3019 and I7A007F1C), the Centro National de Investigaciones Cardiovasculares Carlos III (CNIC) Institutional Animal Research Committee (CNIC 05/13), and the Regional Animal Research Committee (PROEX 51/13). Also, the report of research data from animal experiments follows the guidelines of the ARRIVE (Animals in Research: Reporting In Vivo Experiments) consensus document.^19^

### APC Processing

#### Preparation of hAPCs

The Good Manufacturing Practice–compliant SOP for isolation/expansion of hAPCs has been described previously.^9^ A new investigation was conducted to verify the genetic stability of expanded cell lines. To this aim, we performed (1) chromosome analyses of G‐banded metaphases in hAPCs at the earliest possible passage (P2) and at P6/7 (which is the expansion stage providing sufficient cells for transplantation, without causing replicative senescence)^18^ and (2) microarray comparative genomic hybridization of DNA extracted from vein tissue against DNA extracted from P7 hAPCs.

#### Preparation of sAPCs

Large‐White swine (age 3–4 months) were terminally anesthetized for the collection of peripheral blood (through a jugular vein cannula) and saphenous veins. The vein was processed using a modification of the hAPC‐SOP, by growing single cell suspensions of immunomagnetically sorted CD31^−^CD34^+^ cells onto culture dishes coated with 1% porcine gelatin (Sigma‐Aldrich, UK) containing Endothelial Cell Growth medium‐2 (Lonza, UK) supplemented with 10% porcine serum. Adherent APC colonies appeared after 7 to 10 days of culture and were passaged to new culture dishes once they reached 70% to 80% of confluence. At P2, cells were split for further expansion or generation of frozen stocks. Peripheral blood was used to isolate mononuclear cells by Istopaque‐1077 solution (Sigma‐Aldrich). These cells were processed along with saphenous vein single cell suspensions to verify the selectivity of the modified SOP. The list of donor swine and specific use of corresponding sAPC lines is provided in Table 2.

**Table 2:** Code of Donor Swine and Specific Use of Corresponding sAPC Lines

Swine Code	Line	In Vitro Use	In Vivo Use
020215 A	1	PCR, immunocytochemistry, Flow cytometry, differentiation, ELISA, WB	Efficacy
190515 A	2	PCR, immunocytochemistry, Flow Cytometry, cell size, Matrigel, ELISA, immunogenic activity, WB	Efficacy
190515 B	3	PCR, immunocytochemistry, Flow cytometry, cell size, Matrigel, ELISA, immunogenic activity, WB	Efficacy/engraftment
150615 B	4	Immunocytochemistry, Flow cytometry	
290715 A	5	PCR, immunocytochemistry, Flow cytometry, cell size, differentiation, Matrigel, ELISA, immunogenic activity	Efficacy
220116 A	6	Differentiation, clonogenic assay	
220116 B	7	Differentiation, clonogenic assay, ELISA	

With swine under ketamine/midazolam, blood samples were collected through a cannula inserted into the jugular vein. Animals were then euthanized with Euthatal, and saphenous veins were harvested for isolation and expansion of sAPCs. PCR, polymerase chain reaction; sAPC indicates swine adventitial pericytes; WB, Western blot.

### Assessment of sAPC Features

A series of comparative experiments was performed to assess the purity of cells isolated from swine veins and to verify their similarity to hAPCs. Cells were studied at P5/6, using 4 biological replicates (run in triplicate) unless otherwise specified.

#### Antigenic characteristics

Immunofluorescence microscopy and flow cytometry analyses were performed using the procedures and antibodies described in Tables S1 and S2.

#### Analysis of gene expression

Semiquantitative polymerase chain reaction was performed on cells cultured under normoxia (20% oxygen) or hypoxia (2%) and their conditioned media (CM), using a Quant Studio 6 Flex Real‐Time Polymerase Chain Reaction system (Applied Biosystems). The mRNA expression level was determined using the 2^−ΔCt^ method. The *Taqman* probes used in the molecular biology studies are shown in Table S3.

#### Differentiation and clonogenic assays

Adipogenic and osteogenic differentiation studies were conducted as previously described.^9^ In addition, single‐cell cloning was performed on 2 sAPC lines at P3, using a motorized device connected to the flow cytometric sorter (Cyclone, Beckman Coulter). Sorted cells were placed into each well of a 96‐well culture plate (Greiner Bio‐one, UK) and cultured up to 4 weeks in endothelial cell growth medium‐2 for quantification of colonies generated from a single cell.

#### Analysis of vascular endothelial growth factor A production

The levels of vascular endothelial growth factor A (VEGF‐A) protein were determined in CM by an anti‐human ELISA kit (R&D System, cat n#: DY293B). To this aim, sAPCs (N=3) were cultured in a T25 flask and exposed to normoxia or hypoxia for 48 hours in 2.5‐mL serum‐free, endothelial basal medium 2. In addition, a Western blot analysis was performed to detect the same protein in concentrated CM and unconditioned media (endothelial cell growth medium‐2).

#### Network formation

The capacity of forming networks on Matrigel was assessed using sAPCs or swine pulmonary artery endothelial cells (sPAECs) alone or both in coculture (N=3 biological replicates run in triplicate). In addition, the network formation capacity of sPAECs was assessed following stimulation with sAPC CM or unconditioned media (endothelial cell growth medium‐2).

### Immunogenic Activity of APCs

Studies were carried out to compare the capacity of xenogeneic hAPCs and allogeneic sAPCs (N=3 biological replicates) to induce immune responses upon challenge with swine spleen T lymphocytes.

### In Vivo Transplantation of APCs

#### Study design

Experiments were performed in a total of 42 female Large‐White swine.

**Figure 1:**
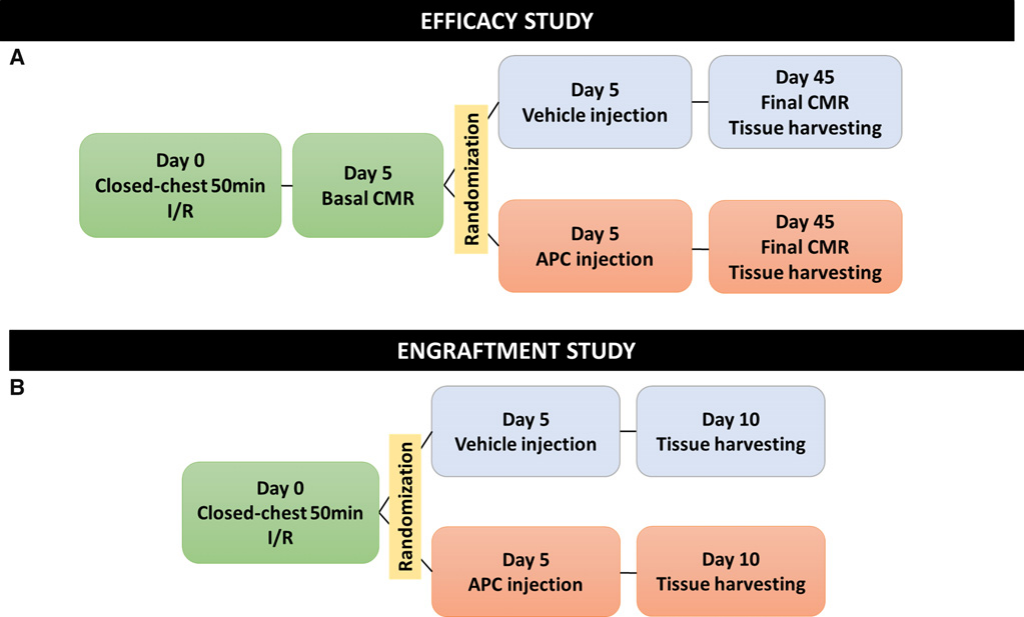
Study design. A, In the efficacy study, swine were subjected to closed‐chest 50‐minute balloon occlusion of the mid‐LAD artery to induce acute MI. At day 5 post‐MI, they underwent a comprehensive basal CMR study. Animals that did not show a transmural infarction (at least 50% of the wall thickness infarcted) were excluded. Immediately after day 5 CMR, animals were randomized to receive intramyocardial vehicle or APC injection via minithoracotomy. CMR was repeated at day 45 post‐MI and hearts were harvested for histology and other tests described in the Materials and Methods section. B, A similar protocol was used to assess cell engraftment with hearts being collected 5 days after vehicle or APC injection. APC indicates adventitial pericyte; CMR, cardiac magnetic resonance; I/R, ischemia/reperfusion; LAD, left anterior descending; MI, myocardial infarction.

A feasibility/efficacy study was conducted in 32 swine according to the protocol summarized in Figure 1A. In brief, reperfused MI was induced at day 0 (vide infra). A cardiac magnetic resonance (CMR) scan was performed 5 days after MI induction, immediately before randomization to intramyocardial injection of vehicle, hAPCs, or sAPCs. A follow‐up CMR scan was performed at 45 days. Immediately after the last CMR scan, animals were euthanized and myocardial tissue samples from the infarct, peri‐infarct, and remote areas were collected for histology, immunohistochemistry, and molecular biology studies.

An additional study was conducted to assess the engraftment of transplanted cells Figure 1B. Eight swine underwent the MI protocol and were then randomized to receive an intramyocardial injection of vehicle or hAPCs. Hearts were collected from animals euthanized at day 10 post‐MI (5 days after vehicle or cell injection) for immunohistochemical assessment of cells expressing the human nuclear antigen and quantitation of human DNA using Alu Element‐Based Polymerase Chain Reaction.^20^ Human and swine GAPDH primers were used as housekeeping genes. Additionally, DNA from cultured hAPCs and swine vascular cells was used as positive and negative controls, respectively.

In a separate experiment, 2 additional swine underwent the same MI protocol and then received an intramyocardial injection of sAPCs, which were labeled 1 day in advance with the long‐term cell tracker CellTrackerTM CM‐DiI (Life Technologies, C700). Briefly, CM‐DiI was diluted 1:1000 in PBS and incubated with confluent cells (adherent to the culture plate) for 5 minutes at 37°C and then at 4°C for an additional 15 minutes, in the dark. Cells were then washed with PBS, fed with fresh media, and placed in the incubator overnight until injection.

### MI Procedure

The protocol to induce acute MI has been described elsewhere.^21^, ^22^ Briefly, the left anterior descending coronary artery, immediately distal to the origin of the first diagonal branch, was occluded for 50 minutes with an angioplasty balloon introduced via the percutaneous femoral route using the Seldinger technique. Balloon location and maintenance of inflation were monitored angiographically. After balloon deflation, a coronary angiogram was recorded to confirm patency of the coronary artery. A continuous infusion of amiodarone (300 mg/h) was maintained during the procedure in all swine to prevent malignant ventricular arrhythmias. A biphasic defibrillator was used to deliver nonsynchronized shocks in case of ventricular fibrillation occurrence.

### CMR Protocol and Analysis

All CMR studies were performed with patients under anesthesia, maintained by continuous intravenous infusion of midazolam, in a 3‐T Achieva Tx whole‐body scanner (Philips Healthcare, Best, The Netherlands) equipped with a 32‐element phased‐array cardiac coil. The protocol included (1) a standard segmented cine steady‐state free‐precession sequence to provide high‐quality anatomical references and to determine left ventricular end‐diastolic wall thickness (EDWT), left ventricular end‐diastolic volume (LVEDV), left ventricular end‐systolic volume (LVESV), and left ventricular ejection fraction (LVEF); (2) a late gadolinium‐enhanced sequence to assess infarct size; and (3) a dynamic acquisition with dual‐saturation technique during gadolinium‐based contrast administration to determine absolute myocardial perfusion.^23^, ^24^ CMR images were processed with a commercial analysis software (QMass MR 7.5 Medis, Leiden, the Netherlands and MR Extended Work Space 2.6, Philips Healthcare) and were analyzed by 2 independent blinded experienced investigators as previously described.^23^, ^24^, ^25^

### APC Transplantation

The expansion of hAPC and sAPC lines in Bristol was synchronized to the schedule for induction of MI in the associate laboratory at the CNIC. Briefly, APCs were expanded to P6, seeded into multilayer flasks (hyperflasks M Cell Culture Vessel, Corning, Cat n#: 10030) allowing cell adhesion for 48 hours, and shipped by a certified courier to reach the CNIC within 24 hours. On the injection day (day 5 post‐MI), APCs were detached by trypsinization and transplanted within 1 hour from suspension in the vehicle, which consisted of endothelial cell growth medium‐2 media used to culture APCs. The choice of injecting APCs at 5 days post‐MI was guided by the consideration that injected cells could perform better if not directly exposed to an acute reperfusion injury.^26^ An aliquot of the suspension was used to assess cell viability by the Trypan Blue exclusion method. An expert cardiac surgeon, blind to the treatment allocation, was responsible for injecting the vehicle or cells in the LV peri‐infarct zone via a left anterior minithoracotomy and direct vision, with animals under general anesthesia. A total of N=10 micro‐injections (for a total volume ≈3 mL) was performed in each animal.

The average injected dose of hAPCs was 21.4×10^6^ cells in the efficacy study (range 16–28×10^6^), and 10.3×10^6^ cells in the engraftment study (range 8–12×10^6^). The dose administered in each single animal is illustrated in Table 3. The efficacy and engraftment studies using sAPCs used the fixed dose of 10×10^6^ cells (Table 4). The rationale for the variable dose of hAPCs is that cell expansion from different veins yield a different number of cells at P6/7, which is largely dependent on the size of the vein leftover. In the case of sAPCs, we used a dose similar to the median injected dose of 10 swine studies reported in a recent meta‐analysis.^8^

**Table 3:** Swine Allocation to Treatments and Dose of hAPC Lines in the In Vivo Studies

Swine ID	Treatment	hAPC Line	No. Injected APCs	Study Completion
Efficacy study				
1900	1	1	28×10^6^	Completed
1901	0	···	···	Completed
1908	Not randomized	···	···	Died at d 1
2389	0	···	···	Completed
2395	0	···	···	Completed
2422	1	2	20×10^6^	Completed
2489	1	3	20×10^6^	Completed
2499	0	···	···	Completed
2569	0	···	···	Died at d 9
2575	1	5	20×10^6^	Completed
2578	1	5	16×10^6^	Completed
2620	1	6	23×10^6^	Completed
2625	Not randomized	···	···	Minor infarct at basal CMR
2626	0	···	···	Completed
2631	1	7+8	22×10^6^	Completed
2637	0	···	···	Completed
2644	0	···	···	Completed
2647	1	6	22×10^6^	Completed
2649	1	7+8	22×10^6^	Completed
Engraftment study				
3265	0	···	···	Completed
3278	0	···	···	Completed
3280	1	10	8×10^6^	Completed
3288	1	11	11×10^6^	Completed
3382	0	···	···	Completed
3383	1	12+13	12×10^6^	Completed
3397	0	···	···	Completed
3400	0	···	···	Completed

Treatment 0, vehicle; Treatment 1, hAPCs (line identification in Table 1). In the efficacy study, 2 animals died before completion of the study: 1 before randomization and 1 in the control group at day 9 post‐MI. In the engraftment experiment, all animals completed the study and were euthanized at 10 days post‐MI. CMR indicates cardiac magnetic resonance; hAPC, human adventitial pericytes.

**Table 4:** Swine Allocation to Treatments and Dose of sAPC Lines in the In Vivo Studies

Swine ID	Treatment	sAPC Line	No. of Injected APCs	Study Completion
Efficacy study				
4117	0	···	···	Completed
4118	1	5	10×10^6^	Completed
4119	0	···	···	Completed
4125	1	2	10×10^6^	Completed
4126	1	3	10×10^6^	Completed
4146	0	···	···	Completed
4147	0	···	···	Completed
4162	0	···	···	Completed
4492	1	5	10×10^6^	Died at d 41
4493	1	3	10×10^6^	Completed
4503	1	2	10×10^6^	Completed
4507	1	1	10×10^6^	Died at d 5
4531	Not randomized	···	···	Died at d 3
Engraftment study				
6075	1	3	10×10^6^	Completed
6080	1	3	10×10^6^	Completed

Treatment 0, Vehicle; Treatment 1, sAPCs (line identification in Table 2). In the efficacy study, 3 animals died before completion: 1 before randomization and 2 in the cell therapy group at day 5 (during immediate surgery recovery) and 41 post‐MI. In the engraftment experiment, all animals completed the study and were euthanized at 10 days post‐MI. MI indicates myocardial infarction; sAPC, swine adventitial pericytes.

Immediately after cell injection, the thoracotomy was closed, and animals were extubated and sent to the recovery room. During their recovery, animals were cared for by dedicated veterinarians and technicians at the CNIC. All animals enrolled in the efficacy studies were returned to the farm before final follow‐up CMR and euthanasia.

### Histological and Immunohistochemistry of Swine Hearts

After a general histological assessment to exclude the presence of tumors or calcification, analyses of microvascular density and fibrosis in peri‐infarct and remote zones were carried out by 2 examiners blind to the treatment allocation, according to methods described previously.^16^, ^17^ Quantitative data from the 2 independent assessments were then averaged and subjected to statistical analyses. Capillaries and arterioles were calculated in 40 fields (at ×200 magnification) in different segments of the myocardium, and the final data were expressed as the number of capillaries or arterioles per mm^2^.

Cardiomyocyte hypertrophy was evaluated by measuring the cell cross‐sectional area in both the ventricular border and remote zones of animals, which received sAPCs or vehicle. After performing antigen retrieval using Citrate Buffer 0.01 mol/L pH=6, for 30 minutes at 98°C, 5‐μm‐thick sections were incubated with Alexa Fluor 488‐conjugated anti‐Wheat Germ Agglutinin (Invitrogen, UK, 1:100, 30 minutes RT), followed by a mouse monoclonal anti‐α‐sarcomeric actin primary antibody (Sigma, UK, 1:200, 2 hours 37°C) and a TRITC‐conjugated goat‐anti mouse IgM secondary antibody (Invitrogen, UK, 1:200, 1 hour Room temperature (RT)). Nuclei were labeled with 4′,6‐diamidino‐2‐phenylindole. Sections were analyzed at ×400 magnification. Images were taken in 1 or 2 sections per sample in areas in which cardiomyocytes were transversally cut. For each sample, cross‐sectional area was measured in 100 cardiomyocytes, in which the nucleus was centrally located within the cell.

Myocardial fibrosis was analyzed by Azan‐Mallory staining followed by morphometric analysis using ImageJ software.^16^ The data were expressed as the percentage of the fibrotic area over the total myocardial area.

In the engraftment studies, the abundance of hAPCs in the peri‐infarct zone was assessed by immunostaining for the human nuclear antigen. We included sections of human hearts as a positive control to make sure the anti‐human antibody was working properly. Immunofluorescence microscopy was used to determine the persistence and location of Dil‐labeled sAPCs in different regions of swine hearts 5 days postinjection. In brief, 5‐μm sections were cut and antigen retrieval was performed after deparaffinization using citrate buffer 0.02 mol/L, pH=6 for 30 minutes at 98°C. Sections were incubated with anti‐isolectin GS‐IB4 (Vector, UK, 1:200, overnight) followed by Alexa Flor 488 Streptavidin secondary antibody (Invitrogen, UK, 1:200, 1 hour, RT). Cardiomyocytes were stained with a mouse anti‐α‐sarcomeric actin (Sigma, UK, 1:200, 2 hours, RT) followed by Alexa Flor 649 anti‐mouse secondary antibody (Invitrogen, UK, 1:200, 1 hour, RT). Nuclei were labeled with DAPI. Sections were analyzed at ×200 magnification with a Zeiss inverted microscope.

Morphometric analyses were performed using the free software ImageJ (http://imagej.nih.gov/ij/).

### Statistical Analysis

All results are presented as mean±SE or range of sample distribution. In some graphs, data are plotted as individual values, with repeated‐measures matched values connected by a line. Gaussian distribution of quantitative values was assessed using the D'Agostino‐Pearson omnibus K2 test. Two‐group comparison analysis was performed by Student *t* test or Mann–Whitney test if data were not normally distributed. For comparison of multiple groups, ANOVA or Kruskal–Wallis test was performed, followed by Holm‐Sidak's or Dunn's multiple comparisons test as appropriate. Correlation between variables was calculated by regression analysis. The contingency Fisher exact test was used to compare the survival rate in the 2 treatment groups. Probability values (*P*) <0.05 were considered significant. Graphs were generated using GraphPad Prism 6 (GraphPad Software, La Jolla, CA).

## Results

### Antigenic Characteristics and Genetic Stability of hAPCs

Using flow cytometry and immunocytochemistry analyses, we confirmed the typical antigenic characteristics of hAPCs reported previously.^9^, ^16^, ^18^ In addition, here we newly assessed the genetic integrity of expanded hAPCs by conventional karyotyping and comparative genomic hybridization arrays. Two out of 5 hAPC lines did not divide in culture sufficiently to allow chromosome analysis. In the remaining 3 lines studied, karyotyping was successful at the expected quality standard both at P2 and P6/7. Two of them showed no evidence of genetic instability. The third cell line showed a balanced rearrangement at P2 and an unrelated unbalanced rearrangement at P7 (Figure S1). As the abnormality detected at P2 was not seen at P7, these are likely to represent random genetic changes occurring in culture, as seen not infrequently in cultured fibroblast cells. Consistently, the comparative genomic hybridization array results were normal and showed no evidence of the deletion detected at P7. The array quality was lower than acceptable for diagnostic testing, which is likely to be because of the quality of DNA extracted from the poorly growing cultures. However, no evidence of copy number change was detected in any region of the genome, which would be considered pathogenic in a diagnostic setting. These data provide reassurance about the biosafety of the expanded human cell product.

### Efficacy Study With hAPCs

#### Clinical outcome

Closed‐chest MI was performed in 19 female Large‐White swine. The dose of cells injected in each swine is reported in Table 3. One swine was excluded from the study randomization because of sudden death within 24 hours after ischemia/reperfusion and another because the animal did not show transmural infarction at day 5 CMR (<50% of wall thickness was infarcted). The remaining 17 swine were randomized to intramyocardial injection of vehicle (N=8) or hAPCs (N=9) immediately after the CMR assessment at day 5 post‐MI. In the control group, 1 swine died 4 days after the injection of vehicle. Thus, 16 swine (N=7 given vehicle and N=9 given hAPCs) completed the study. The overall mortality rate for this set of experiments was 11%. Contingency square analysis by Fisher exact test indicates there was no difference between the 2 groups regarding survival (2‐tailed *P*=0.47). There was no difference in body weight or hematocrit between groups at day 5 and day 45 (Table 5). Likewise, there was no effect of cell therapy on body weight gain from day 5 to day 45 (18.1±1.4 kg versus 18.6±1.4 kg in the vehicle group).

**Table 5:** CMR Parameters of the hAPC Efficacy Study

Parameter	Control Group			Cell Group		
	Day 5	Day 45	Delta	Day 5	Day 45	Delta
BW, kg	32.6±1.4	51.2±2.3†	18.6	34.8±0.7	52.9±1.7†	18.1
Ht, %	30.4±0.6	31.6±0.6	1.2	29.2±1.1	31.2±0.5	2.0
LV mass, g/m^2^	80.9±5.6	78.4.±2.7	−2.5	85.9±4.2	84.5±2.2	−1.4
Infarct size (% LV)	33.0±3.2	22.7±2.4†	−10.3	33.3±2.9	23.4.±2.6†	−9.9
EDWT, infarct—mm	5.7±0.9	4.0±0.4†	−1.7	6.0±0.9	4.4±0.8†	−1.7
EDWT, remote—mm	4.5±0.8	5.9±0.6†	1.4	4.8±0.7	5.6±0.4†	0.8
LVEDV, mL/m^2^	157.3±5.1	179.6±8.9*	22.3	159.4±4.3	192.8±6.8†	33.4
LVESV, mL/m^2^	92.7±5.2	114.1±10.9	21.4	94.3±2.7	120.8±9.0*	26.5
LVEF, %	41.2±2.4	37.2±3.5	−4.0	40.7±1.3	38.0±2.6	−2.7
Infarct core perfusion, mL/100 g per min		95.9±21.7			87.2±17.5	
Anterior border zone perfusion, mL/100 g per min		84.7±8.2			80.2±16.2	
Inferior border zone perfusion, mL/100 g per min		109.2±6.5			101.5±9.7	
Remote zone perfusion, mL/100 g per min		129.4±8.5			126.0±8.8	

Data are presented as mean±SE. N=7 and 9 swine in control and cell therapy group, respectively. CMR perfusion was not assessed in 1 swine in control and 1 swine in the cell group at day 45 because of technical issues. Therefore, quantitative myocardial perfusion was assessed in 6 and 8 swine at day 45, respectively. BW indicates body weight; CMR, cardiac magnetic resonance; EDWT, end‐diastolic wall thickness; hAPCs, human adventitial pericytes; Ht, hematocrit; I/R, ischemia/reperfusion; LV, left ventricular; LVEDV, LV end diastolic volume; LVEF, LV ejection fraction; LVESV, LV end‐systolic volume.

*P<0.05 and ^†^ *P*<0.01 vs baseline (day 5 after I/R).

#### CMR outcome

**Figure 2:**
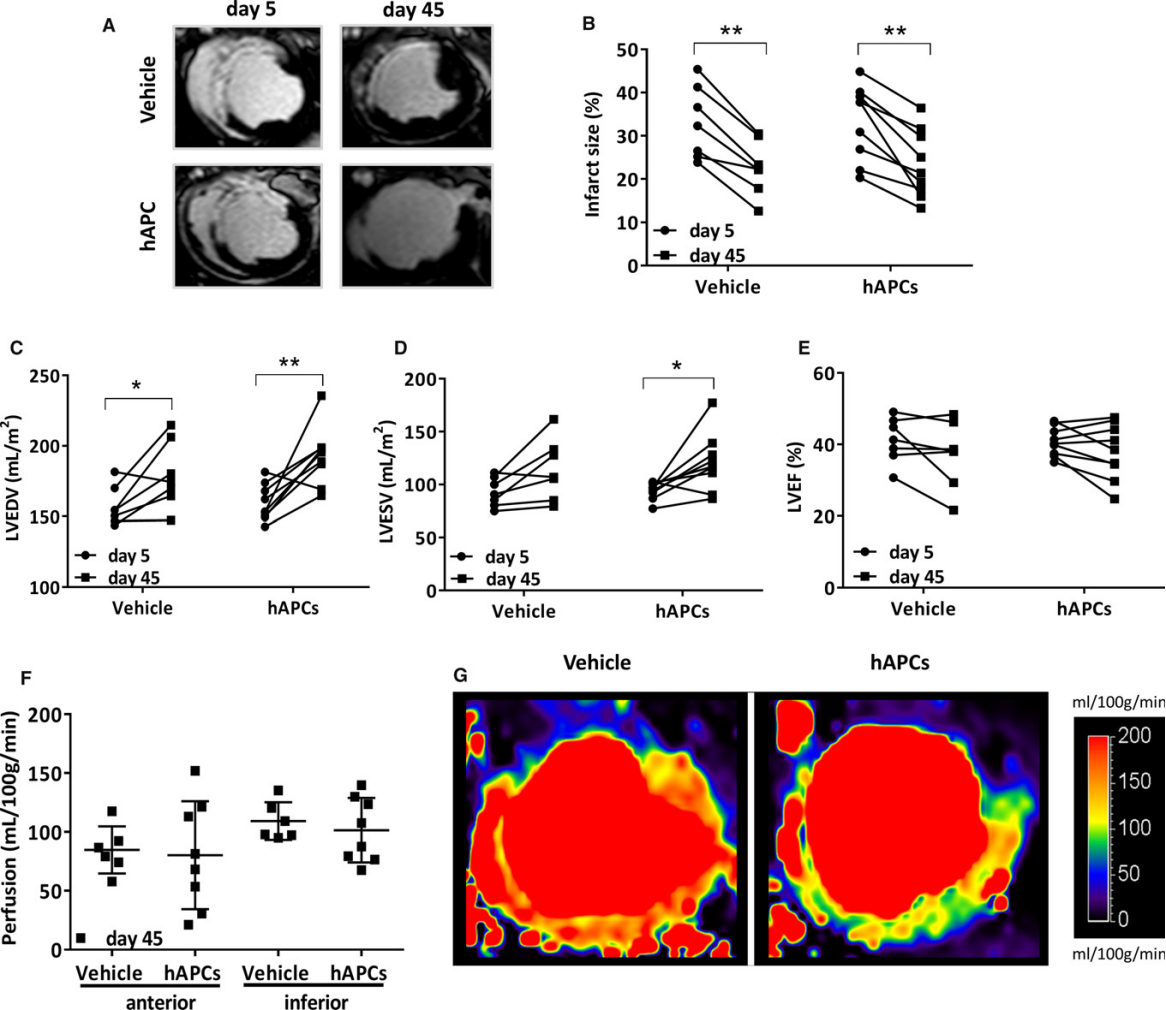
Cardiac parameters of individual animals measured by CMR at day 5 (prior injection of vehicle or hAPCs) and day 45 post‐MI (end of the study). A, Representative late gadolinium enhancement images at day 5 and day 45 post‐MI in 2 swine injected with vehicle or hAPCs (donor human cell line ID# 6). B through F, Plot individual data of infarct size (B), LVEDV (C), LVESV (D) both normalized for body surface area, LVEF (E), and perfusion in the anterior and inferior border zones at day 45 post‐MI (F). G, Representative myocardial perfusion maps at day 45 post‐MI in 2 swine injected with vehicle or hAPCs (donor human cell line ID# 6). Perfusion maps were scaled between 0 and 200 mL/100 g per min. *P<0.05 and **P<0.01 vs day 5. CMR indicates cardiac magnetic resonance; hAPCs, human adventitial pericytes; ID, identity number; LVEDV, left ventricular end diastolic volume; LVEF, left ventricular ejection fraction; LVESV, left ventricular end systolic volume; MI, myocardial infarction.

Table 5 shows the relevant parameters that were measured at day 5 post‐MI (before injection of vehicle or hAPCs) and day 45 post‐MI as well as the related absolute changes from day 5 to day 45. Representative CMR videos are reported as Videos S1 through S4. Both groups were well balanced for basal measurements, and there was no difference between treatments at baseline and terminal assessment about the infarct size (Figure 2A and 2B), LV volumes (Figure 2C and 2D), and LVEF (Figure 2E) (all *P*>0.25). Likewise, no differences were detected in perfusion at day 45 in all the evaluated segments (Figure 2F, *P*>0.15). Representative images of CMR perfusion maps are shown in Figure 2G.

#### Histological outcome

**Figure 3:**
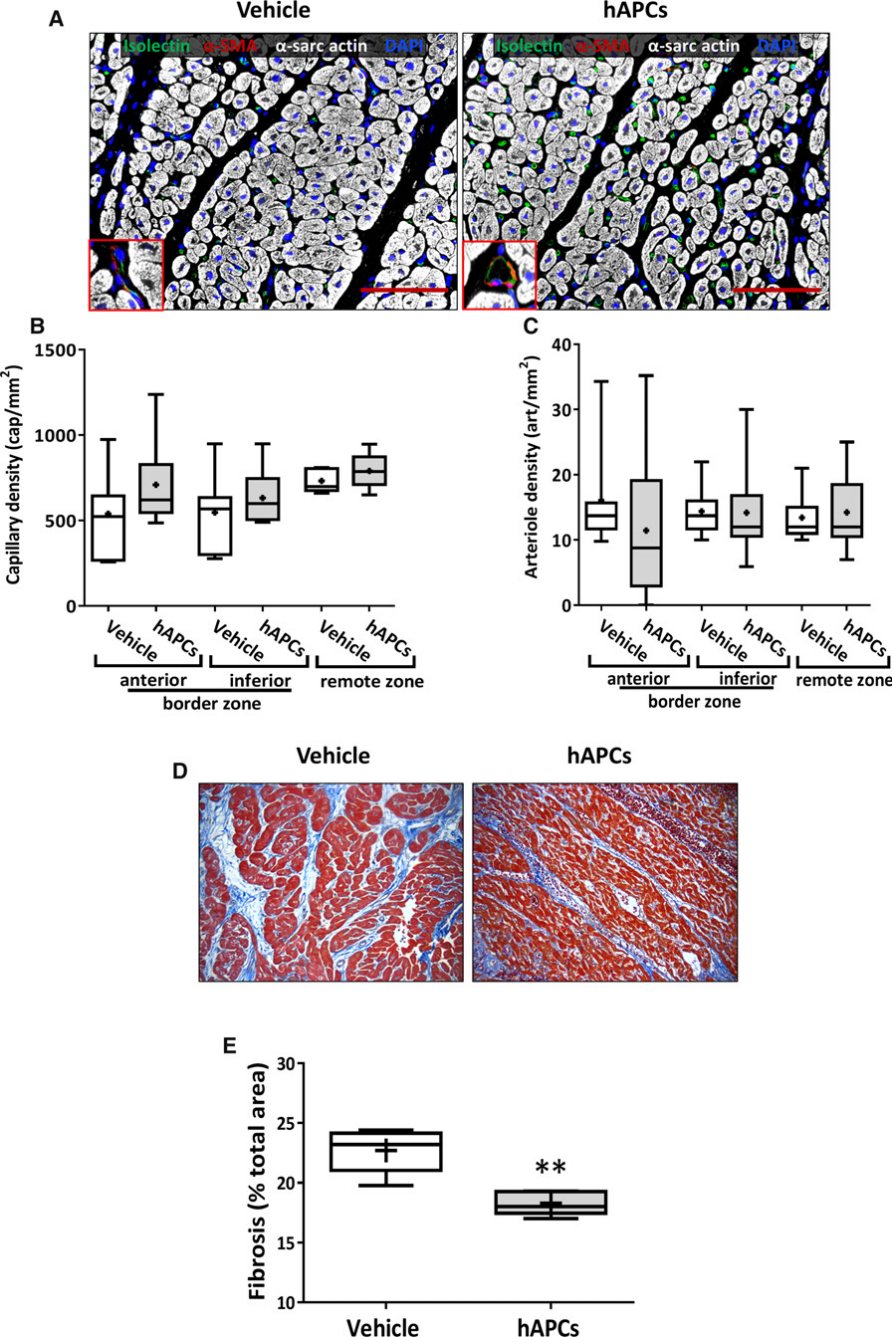
Immunohistochemistry analysis of hearts collected at day 45 post‐MI from swine injected with vehicle or hAPCs. A, Representative confocal images of capillaries and arterioles in infarcted hearts injected with vehicle or hAPCs (donor human cell line ID# 6). Vessels are stained with isolectin GS‐IB4 (green) and α‐SMA (red); cardiomyocytes are stained with α‐sarc actin (white) and nuclei with DAPI (blue); Inserts show α‐SMA positive arterioles. Scale bar=100 μm. B and C, Graphs show capillary density (B) and arteriole density data (C). Values were not normally distributed. The bottom and top of the boxes represent the first and third quartiles, while whiskers indicate the 5 to 95 percentile. Median and mean values are represented as a horizontal line and “+” symbol inside the boxes, respectively. D and E, Analysis of fibrosis in the infarct border‐zone. Representative immunohistochemistry image of Azan‐Mallory (total optical magnification ×200) (D) and graphs showing the quantification of fibrosis in hearts injected with vehicle or hAPCs (donor human cell line ID# 6). E, **P<0.01 vs vehicle group. α‐SMA indicates α‐smooth muscle actin; α‐sarc actin, α‐sarcomeric actin; DAPI, 4′,6‐diamidino‐2‐phenylindole; hAPCs, human adventitial pericytes; ID, identity number.

General examination excluded the occurrence of malignant degeneration or calcification in hearts from the 2 treatment groups. In addition, there was no difference between treatments as far as the capillary or arteriole density end point is concerned (*P*>0.20 for both comparisons, Figure 3A through 3C). Importantly, hAPC therapy reduced interstitial fibrosis in the infarct border zone (−19% compared with the vehicle, *P*<0.01, Figure 3D and 3E), whereas the infarct scar was unaffected.

### Engraftment Study With hAPCs

Eight swine underwent the MI protocol and were randomized 5 days later to intramyocardial injection of vehicle (N=5) or hAPCs (N=3). The dose of cells injected in each swine is reported in Table 3. No casualties occurred in this set of experiments. Immunohistochemistry studies of hearts collected at day 10 post‐MI did not detect human cells in any treatment group. These data were validated and confirmed by real‐time polymerase chain reaction–based quantitation of human DNA. In parallel experiments, we could detect the human‐specific Alu sequence in the genomic DNA mixture from hAPCs but not in the extracts of hAPC‐ or vehicle‐injected swine hearts. Results suggest a rapid clearance of the injected xenogeneic cells, in contrast to the reportedly long‐lasting persistence of hAPCs following injection into mouse infarcted hearts.^16^

### Immunogenic Activity of hAPCs

**Figure 4:**
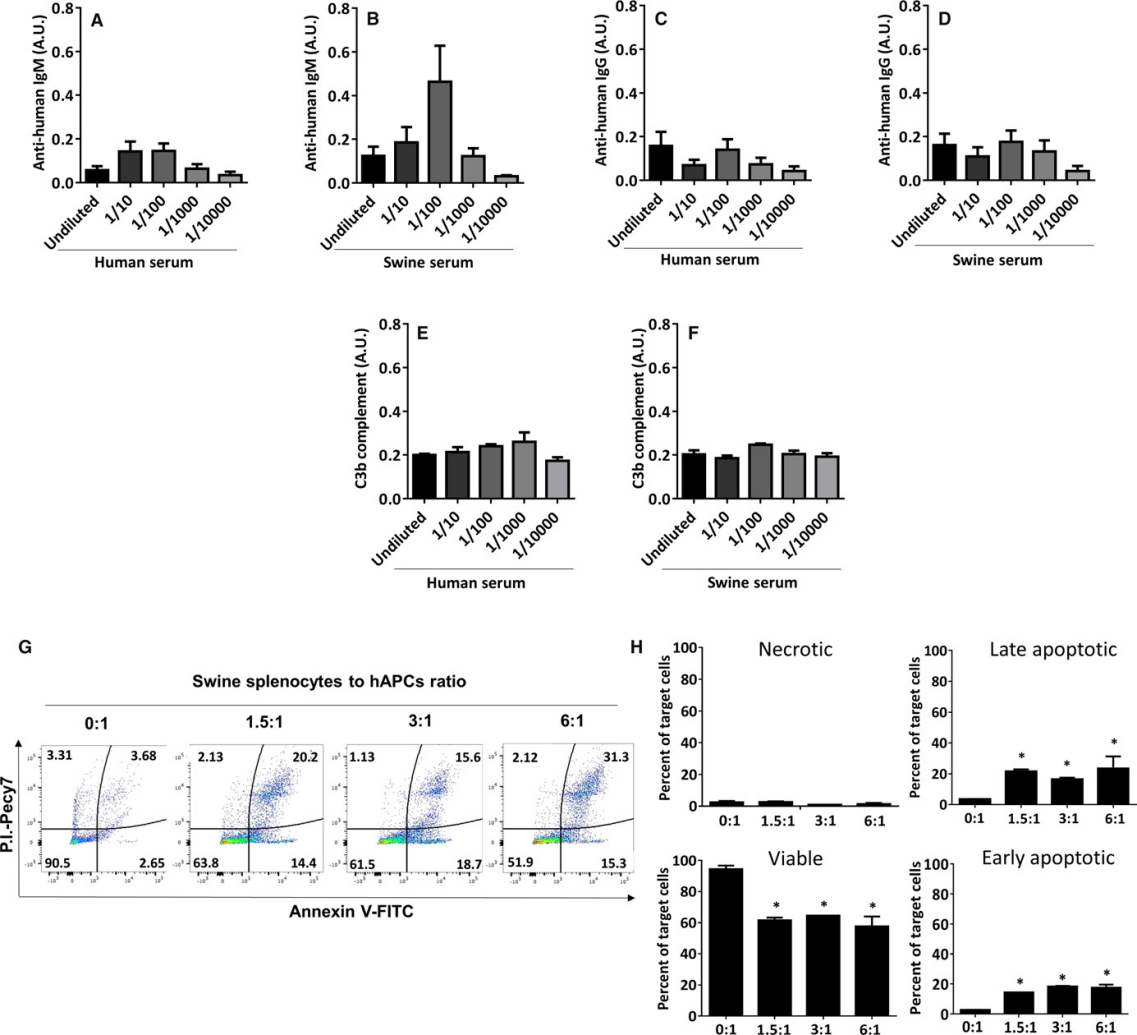
Assessment of swine immune response to hAPC. A through F, Humoral immune response to hAPCs. hAPCs were incubated with swine or human serum (negative control) at different dilutions. There was no difference in the binding of anti‐swine IgM or IgG antibodies on hAPCs when incubated with human serum (A and C) or swine serum (B and D). Similarly, no difference was observed with regard to the levels of C3b, a metabolite of complement cascade (E and F). Data are expressed as mean±SEM; N=3 biological replicates. G and H, Cytotoxic activity of swine splenocytes on hAPCs. To investigate cell‐mediated immune response, hAPCs (target cells) were incubated with interleukin‐activated swine splenocytes (effector cells). G, Representative flow cytometry graphs of hAPCs challenged with increasing concentrations of swine splenocytes. Values in each quadrant represent the percent of total cells. H, Bar graphs show the percent of necrotic (Annexin V^−^/P.I.^+^), late apoptotic (Annexin V^+^/P.I.^+^), early apoptotic (Annexin V^+^/P.I.^−^), and viable (Annexin V^−^/P.I.^−^) hAPCs. Data are expressed as mean±SEM; N=2 biological replicates; *P<0.05 vs the “0:1” group. A.U. indicates Arbitrary Units; FITC, Fluorescein isothiocyanate; hAPCs, human adventitial pericytes; P.I., propidium iodide.

Our previous work in mice suggests that hAPCs are immune privileged, thereby enabling transplantation across major histocompatibility barriers.^16^ Here, we verified this possibility by assessing the humoral and cell‐mediated immune responses in swine. Results of 3 separate experiments showed the absence of natural antibodies against hAPCs in pre‐immune porcine serum (Figure 4A through 4D). Additionally, we could not observe any complement activation as assessed by ELISA of complement component 3b (Figure 4E and 4F) or complement‐dependent cytotoxicity (caspase activity, data not shown). However, hAPCs triggered a strong immune reaction by swine splenocytes (Figure 4G and 4H). In line, immunohistochemistry of swine hearts injected with hAPCs showed a 2‐fold higher infiltration of CD45/CD3 lymphocytes as compared with vehicle‐injected hearts (data not shown).

### Isolation, Expansion, and Characterization of sAPCs

**Figure 5:**
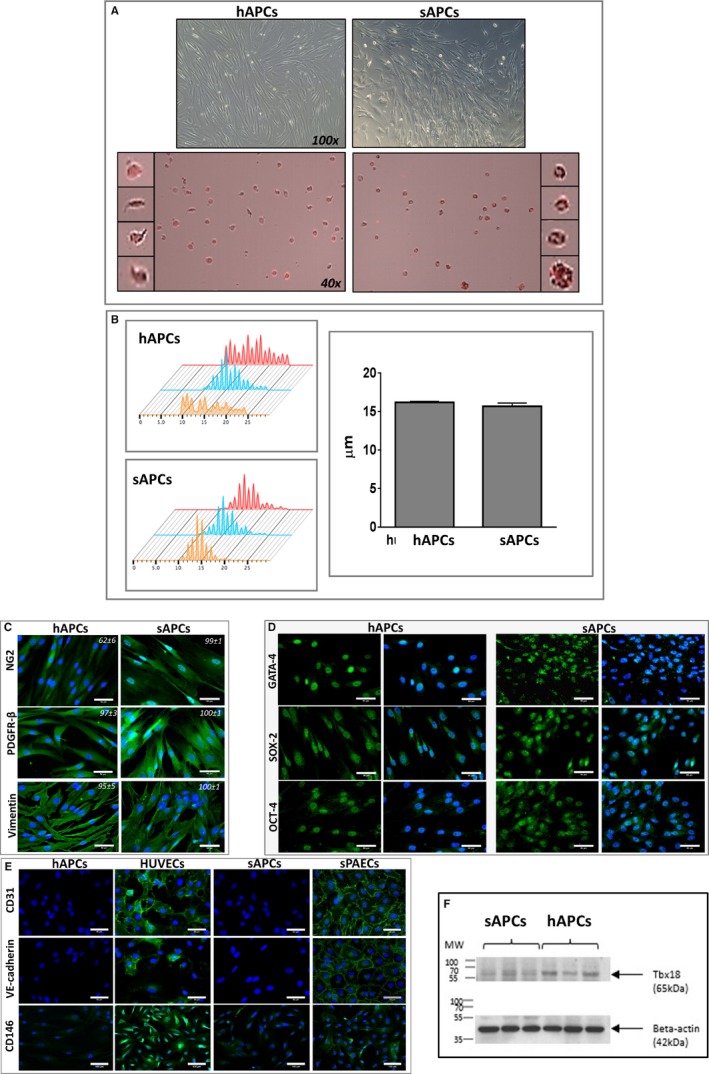
Comparison of APCs isolated from human and swine saphenous veins. A, Upper panels: phase contrast microscopy images of human and swine cells displaying similar spindle‐shape features (magnification ×100). Lower panels: Cells shown in the contrast‐phase microscopy images are stained with swine and human PE CD105 antibodies (PE=red laser) for measurement of cell size by Tali Image‐based cytometer (magnification ×40). B, Cell size histograms calculated by Tali and Novocyte 3000 (Acea, Biosciences, Inc). The *X* axis in the left panels represents the cell size and the *Y* axis represents the number of cells counted (3 cell lines for each group). The bar graph shows the mean and SEM, which was similar between groups, though the range of cell size was wider for hAPCs. C, Representative immunofluorescence microscopy images show both hAPCs and sAPCs express the typical mesenchymal markers NG2, PDGFR‐β, and Vimentin. Values in each panel represent the mean±SEM of 4 biological replicates. D, Immunofluorescence microscopy images show the expression of cardiac transcriptional factor GATA‐4, and the stemness markers OCT‐4 and SOX‐2. Blue fluorescence of DAPI recognizes nuclei. Magnification ×200 and ×400 (50‐μm scale bar). E, Representative immunofluorescence microscopy images of hAPCs and sAPCs confirming these cells do not express endothelial antigens, at variance with HUVECs and PAECs (positive controls). F, Western blot image showing Tbx18 protein corresponding to the 65 kDa MW within human and swine APC lysate. APCs indicates adventitial pericytes; DAPI, 4′,6‐diamidino‐2‐phenylindole; GATA‐4, GATA binding protein 4; hAPCs, human adventitial pericytes; HUVECs, human umbilical vein endothelial cells; MW, molecular weight; sPAECs, swine pulmonary artery endothelial cells; PDGFR‐β, platelet‐derived growth factor receptor‐β; OCT‐4, octamer‐binding transcription factor 4; sAPCs, swine adventitial pericytes; SOX‐2, sex determining region Y‐box 2.

Having demonstrated that the swine is not immune tolerant to hAPCs and therefore not ideal for studying their therapeutic activity, we next evaluated whether an equivalent cell product could be obtained from swine saphenous veins. To this aim, we adapted the SOP previously used for isolation/expansion of hAPCs, with main modifications being the substitution of fetal bovine serum with swine serum and use of swine gelatin as a coating material of culture dishes. We expanded cells from 7 swine veins for this study (with a success of 100%). Cells grew quickly in culture with an average doubling time of 2.5 days, reaching ≈30 million at P6 in 6 to 8 weeks. Both saphenous vein–derived swine cells and hAPCs showed similar spindle‐shape morphology at contrast phase microscopy (Figure 5A) and sizes as assessed by an image‐based cytometer (Figure 5B).

We next performed immunohistochemistry and flow cytometry studies to compare the 2 populations, using validated species‐specific antibodies as indicated in Tables S1 and S2. The similarity of the 2 cell products was confirmed by immunocytochemistry analyses showing equivalent high expression of the lineage antigens NG2, PDGFRβ, and vimentin (Figure 5C) and of the stemness markers GATA‐4, sex‐determining region Y‐box 2 (SOX‐2), and octamer‐binding transcription factor 4 (Figure 5D). Both saphenous vein–derived swine cells and hAPCs were negative for the endothelial markers CD31 and VE‐cadherin, and expressed very low levels of CD146, whereas human umbilical vein endothelial cells and sPAECs (positive controls) express all the above antigens (Figure 5E). We also verify the expression of the T‐box family transcription factor Tbx18, which is reportedly selectively expressed by microvascular pericytes and vascular smooth muscle cells in the adult mouse.^27^ Results confirm that the *TBX18* gene is detected by polymerase chain reaction for both cell lines (Ct value hAPCs=24.4 and Ct value sAPCs=26.6, data not shown), with the expression being confirmed at the protein level by Western blot analyses of cell lysates (Figure 5F).

**Figure 6:**
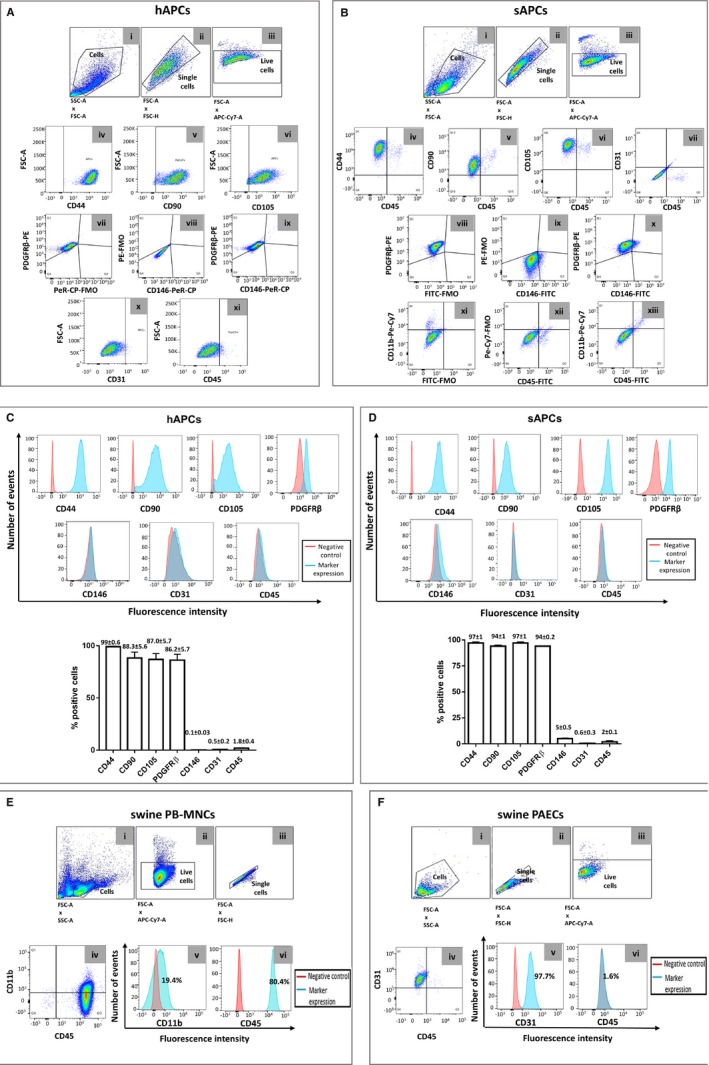
Flow cytometry analyses of APCs isolated from human and swine saphenous veins. A and B, Representative flow cytometry gating procedure of hAPC line #1 and sAPC line #1 at P5. Total cell populations and the single cells (singlets) were gated according to FSC‐A vs SSC‐A and FSC‐A vs FSC‐H parameters (i and ii). Viable cells were distinguished from dead cells using Fixable Viability Dye eFluor780 (iii) and further gated for selected antigens (iv through xi and iv through xiii). Pericyte, mesenchymal, endothelial, and hematopoietic markers were studied. The FMO control was used in the assessment and gating of CD146^+^ and PDGFRβ^+^ cells, because of the use of multiple fluorochromes (vii through ix and viii through x). The same approach was used when studying the expression of CD45 and CD11b on sAPCs to exclude hematopoietic cell contamination in the cell culture system (xi through xiii). Data were acquired using FACSCantoII (BD Biosciences) or Novocyte 3000 flow cytometer (ACEA Biosciences, San Diego, CA, USA) and analyzed using the FlowJo v10.3 software. C and D, Flow cytometry histograms for each surface marker in representative hAPC (C) and sAPC lines (D). Negative control staining profiles are shown by the red histograms, whereas specific antibody staining profiles are shown by light blue histograms. Bar graphs show the mean±SEM values of 3 hAPC and sAPC lines. E and F, Gating and histograms of fresh isolated swine PB‐MNCs and swine PAEC line #1 at P5 used as positive control for the staining of hematopoietic and endothelial markers, respectively. In both cell lines, the negative control staining profile is shown by full red histogram, while the positive staining profile is shown by full light‐blue histogram. FSC‐A indicates Forward Scatter Area; FSC‐H, Forward Scatter Height; FMO, fluorescence minus 1; hAPCs, human adventitial pericytes; PB‐MNCs, Peripheral blood mononuclear cells; PDGFRβ, platelet‐derived growth factor receptor‐β; sAPCs, swine adventitial pericytes.

Furthermore, flow cytometry studies indicate that, similar to hAPCs, saphenous vein–derived swine cells abundantly express the mesenchymal markers CD44, CD90, CD105, and PDGFRβ but are negative for CD146, CD31, and the hematopoietic marker CD45 (Figure 6A through 6D). Swine peripheral blood mononuclear cells and PAECs were used as positive controls (Figure 6E and 6F). In addition, experiments were conducted to exclude the possibility that contamination of the vein extracts by circulating hematopoietic cells may compromise the purity of the cell product. To this aim, we isolated mononuclear cells from swine peripheral blood by Histpaque‐1077 solution and cultured them using the above‐described APC‐SOP, with or without initial immune‐magnetic beads sorting for CD34/CD31. In both cases, no cell emerged during a 1‐month‐long culture, thus providing a definitive confirmation that the adopted SOP is selective. Altogether these data indicate that the expanded swine cell product is a bona fide equivalent of hAPCs.

### Clonogenic and Differentiation Potential of sAPCs

Next, we attempted to confirm the equivalence on functional grounds. To investigate whether sAPCs can form clones, total cells from 2 biological replicates were sorted as single cells and cultured in 96‐well plates. Two weeks later, 23% of cells from 1 sAPC line formed colonies (57 out of 240), while the other APC line gave rise to 67 colonies (27%). Of these primary colonies, 9 and 14 could be further expanded in culture.

We next exposed 3 sAPC lines to differentiation stimuli for the derivation of mesenchymal lineages. All the lines showed typical differentiation capacities, as denoted by the accumulation of fat droplets and Oil‐Red‐O staining in the adipogenesis assays and Alizarin Red and Alkaline phosphatase staining in the osteogenesis assays (Figure S2). These results denote similarities with the reported clonogenic activity and in vitro differentiation properties of hAPCs.^9^

### Proangiogenic Activity of sAPCs

**Figure 7:**
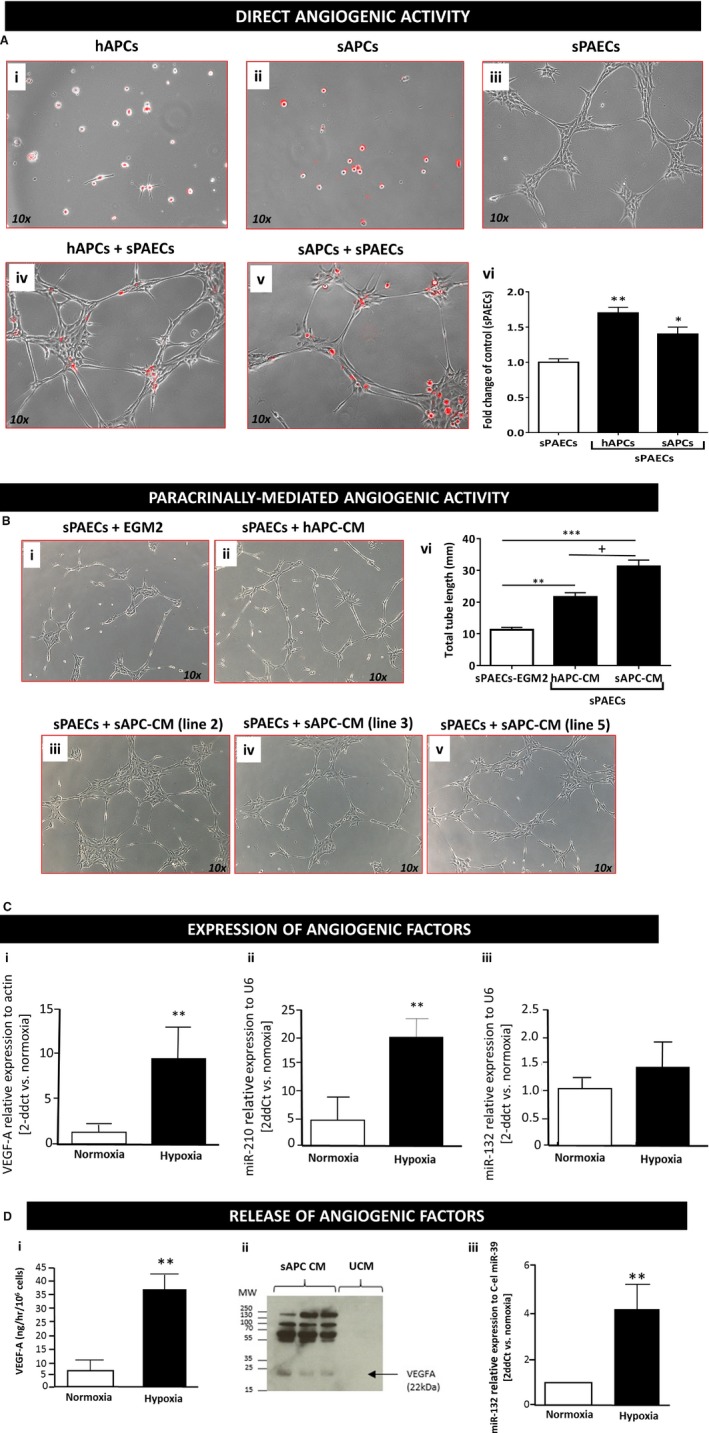
A, Representative phase‐contrast and fluorescent images of hAPCs (Ai), sAPCs (Aii), and sPAECs (Aiii) cultured alone or in combination (Aiv, hAPCs+sPAECs; Av, sAPCs+sPAECs at a 1:4 ratio). APCs are stained with the long‐term cell tracker Dil (red fluorescence). (Avi) Bar graph showing the fold increase in cumulative tube length induced by coculturing APCs with sPAECs in 2 separate experiments, each comprising 3 APC lines per group. Data are represented as mean±SEM, *P<0.05 and **P<0.01 vs sPAECs. B, Representative phase‐contrast images of sPAEC networks formed on Matrigel in the presence of EGM2 medium (Bi) or human (Bii) or swine APC‐CM (Biii‐v, 3 cell lines). Magnification ×10. (Bvi) Histograms summarize quantitative data of the tube length per field in an experiment comprising 3 APC lines per group). Data are represented as mean±SEM, **P<0.01 and ***P<0.001 vs PAECs. ^+^ *P*<0.05 vs hAPC‐CM. C, Bar graphs shows the expression of VEGF‐A (i), miR‐210 (ii), and miR‐132 (iii) in sAPC lysate under normoxia and following stimulation by hypoxia. D, VEGF‐A (i) and miR‐132 (iii) were also found in conditioned media and increased following hypoxia. Western blot image displaying secreted VEGF‐A (MW=22 kDa) and also larger bands under normoxia condition (ii). The antibody detection of these bands suggests that either multiple molecules are bound together in a multimerized complex or that VEGF‐A is bound to another molecule with the region bound by the antibody exposed/separate from the region bound by the corresponding molecule/receptor. Values are means±SEM. **P<0.01 vs normoxia. CM indicates conditioned medium; Dil, 1,1′‐Dioctadecyl‐3,3,3′,3′‐Tetramethylindocarbocyanine; hAPCs, human adventitial pericytes; miR, microRNA; MW, molecular weight; sAPCs, swine adventitial pericytes; sPAECs, swine pulmonary artery endothelial cells; VEGF‐A, vascular endothelial growth factor A.

One important feature of hAPCs consists of their ability to modulate angiogenesis via direct contacts with, and transfer of paracrine growth factors and microRNAs to endothelial cells.^9^, ^16^, ^28^ Therefore, we next investigated whether sAPCs share similar properties by testing their capacity to promote endothelial cell network formation in a Matrigel assay. Interestingly, preliminary experiments showed that the sAPC‐CM potentiates the network‐forming capacity of human umbilical vein endothelial cells, whereas the direct contact of sAPCs and human umbilical vein endothelial cells in coculture inhibits the network formation (data not shown). This suggests that the physical interaction between cocultured xenogeneic cells can override the proangiogenic activity of the sAPC secretome. To exclude any species‐specific incompatibility that may confound the interpretation of results, we next used swine endothelial cells (ie, sPAECs) as an angiogenic target for sAPCs. In this setup, both sAPCs (Figure 7A) and their CM (Figure 7B) led to potentiation of the network‐forming capacity of sPAECs. Moreover, the sAPC secretome exhibited superior proangiogenic activity as compared with the hAPC secretome (Figure 7B), with this difference being compatible with a higher specificity of the sAPC‐derived paracrine factors for sPAECs.

### Angiocrine Properties of sAPCs

Our previous studies showed that hAPCs express and secrete a spectrum of angiogenic factors and microRNAs.^9^, ^16^, ^17^ Likewise, as shown in Figure 7C and 7D, sAPCs express and secrete VEGF‐A and the proangiogenic miR‐132. They also express the hypoxia‐inducible miR‐210, with levels being increased in response to hypoxia (Figure 7C).

### Immunogenic Activity of sAPCs

**Figure 8:**
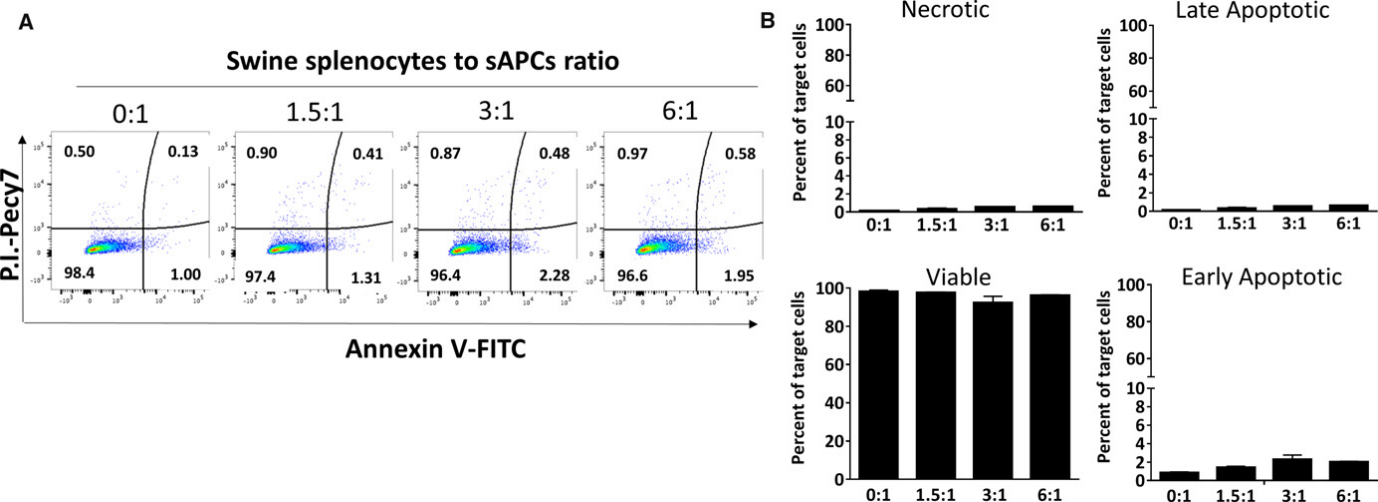
Cytotoxic assay of sAPCs and swine splenocytes. To investigate the cell‐mediated immune response, sAPCs (target cells) were incubated with interleukin‐activated swine splenocytes (effector cells). A, Representative scattergram of sAPCs challenged with increasing concentrations of swine splenocytes. Values in each quadrant represent the percent of total cells. B, Bar graph showing the percent of necrotic (Annexin V^−^/P.I.^+^), late apoptotic (Annexin V^+^/P.I.^+^), early apoptotic (Annexin V^+^/P.I.^−^), and viable (Annexin V^−^/P.I.^−^) sAPCs. Data are expressed as mean±SEM; N=3 biological replicates. FITC indicates Fluorescein isothiocyanate; P.I., propidium iodide; sAPCs, swine adventitial pericytes.

We performed a cytotoxic assay where sAPCs were challenged with interleukin‐activated swine splenocytes. An initial assay with cells in suspension showed no cytotoxic effect. However, results were confounded by the sAPC tendency to undergo spontaneous, time‐dependent apoptosis (Figure S3). Therefore, we repeated the assay with cells in adhesion. Results confirmed the compatibility between allogeneic sAPCs and effector immune swine cells (Figure 8).

### Efficacy Study With sAPCs

#### Clinical outcome

Closed‐chest reperfused MI was induced in 13 female Large‐White swine. Table 4 illustrates the attribution of swine to the 2 arms of the study, the cell line code and dose, and the study outcome for each animal. One swine was excluded from the study randomization because of sudden death at day 3 after MI. The remaining 12 swine were randomized to intramyocardial injection of vehicle (N=5) or sAPCs (N=7) after the CMR assessment at day 5 post‐MI. In the sAPC group, 1 swine died within a few hours after cell transplantation and another one at 1 week before the 45‐day post‐MI follow‐up. Thus, 10 swine (N=5 given vehicle and N=5 given sAPCs) survived completing the full protocol. The overall mortality rate for this set of experiments was ≈23%. Contingency square analysis indicates there was no difference between the 2 groups for survival (*P*=0.46). Furthermore, the cumulative analysis of survival in swine given cell therapy with hAPCs and sAPCs (n=14 out of 16) or vehicle (n=12 out of 13) showed no difference between groups (2‐tailed *P*=1.00). There was no difference in body weight or hematocrit between groups at day 5 and day 45 (Table 6). Likewise, there was no effect of sAPC therapy on body weight gain from day 5 to day 45 (11.5±1.8 kg versus 10.9±1.4 kg in the vehicle group).

**Table 6:** CMR Parameters of the sAPC Therapy Study

Parameter	Control Group			Cell Group		
	Day 5	Day 45	Delta	Day 5	Day 45	Delta
BW, kg	35.1±2.0	46.0±1.5†	10.9	31.5±0.6	43.0±1.7†	11.5
Ht, %	28.7±0.7	31.5±0.7†	2.8	29.2±0.8	33.5±1.1*	4.2
LV mass, g/m^2^	78.1±4.7	83.6±4.9	5.5	73.2±3.2	81.5±6.3	8.3
Infarct size (% LV)	36.9±4.7	23.9±1.7*	−13.0	35.1±2.9	24.3.±2.6*	−10.8
EDWT, infarct—mm	5.8±1.0	3.8±0.6†	−2.0	4.9±0.5	4.0±0.8†	−1.0
EDWT, remote—mm	4.2±0.3	5.3±0.8†	1.1	3.8±0.3	5.2±0.8†	1.4
LVEDV, mL/m^2^	155.8±8.6	188.0±13.5†	32.3	159.9±5.5	193.0±11.7*	33.1
LVESV, mL/m^2^	93.3±7.2	114.9±12.0*	21.6	99.6±6.9	124.1±14.1*	24.5
LVEF, %	40.3±2.3	39.5±2.7	−0.8	37.9±2.8	36.4±4.1	−1.5
Infarct core perfusion, mL/100 g per min		98.1±13.8			91.8±14.8	
Ant border zone perfusion, mL/100 g per min		122.9±18.6			102.6±18.9	
Inf border zone perfusion, mL/100 g per min		115.3±12.5			129.9±26.1	
Remote zone perfusion, mL/100 g per min		118.2±9.8			116.4±22.7	

Data are presented as mean±SE. Ant indicates anterior; BW, body weight; CMR, cardiac magnetic resonance; EDWT, end‐diastolic wall thickness; Ht, hematocrit; Inf, inferior; I/R, ischemia/reperfusion; LV, left ventricular; LVEDV, LV end diastolic volume; LVEF, LV ejection fraction; LVESV, LV end‐systolic volume; sAPCs, swine adventitial pericytes.

*P<0.05 and ^†^ *P*<0.01 vs baseline (day 5 after I/R). N=5 and 5 swine in control and cell therapy groups, respectively.

#### CMR outcome

**Figure 9:**
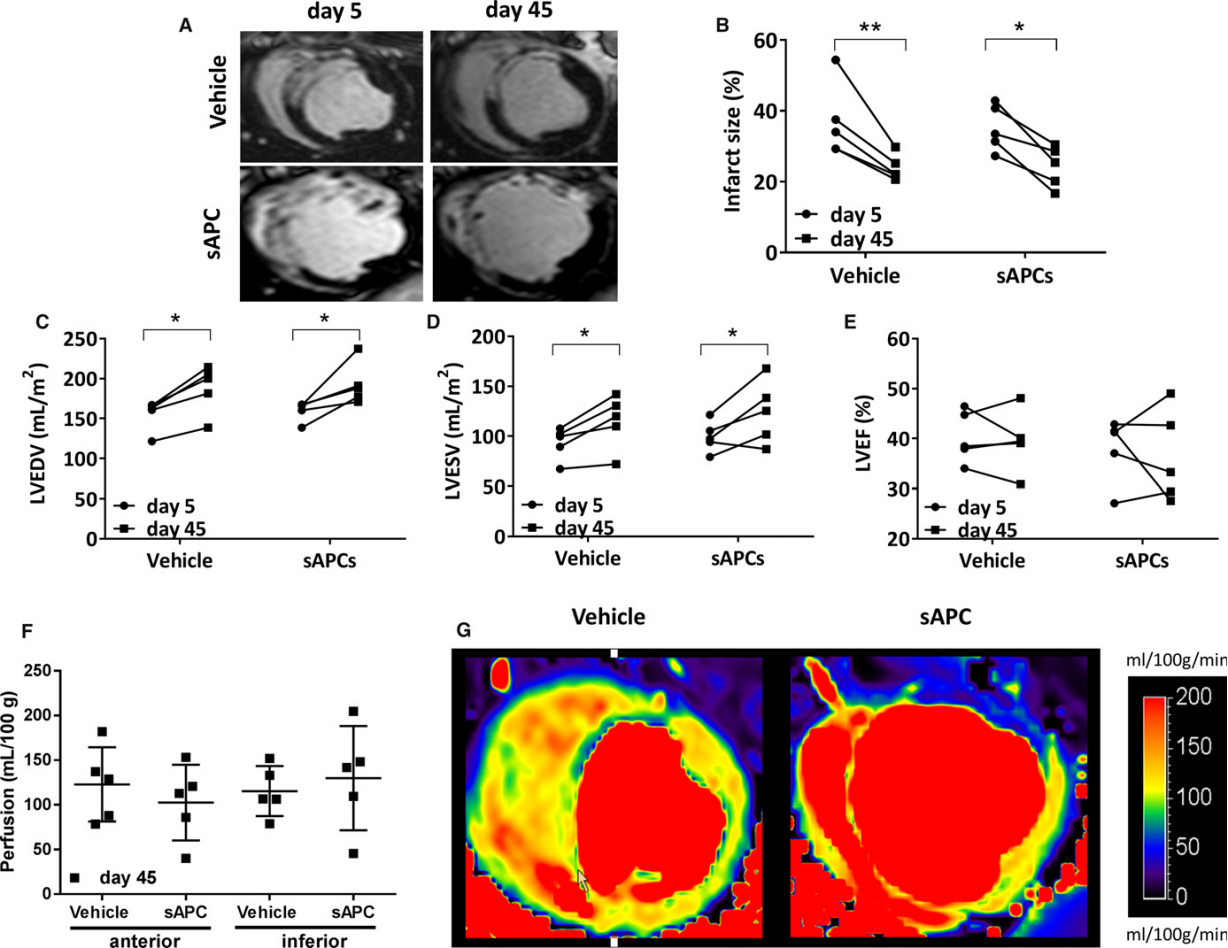
Cardiac parameters of individual animals measured with CMR at day 5 (prior injection of vehicle or sAPCs) and day 45 post‐MI (end of the study). A, Representative late gadolinium enhancement images at day 5 and day 45 post‐MI in 2 pigs injected with vehicle or sAPCs (donor swine cell line ID# 3). B through E, Plot individual data of infarct size (B), LVEDV (C), LVESV (D), and LVEF (E). F and G, Perfusion in the anterior and inferior border zones at day 45 post‐MI. Individual data (F) and representative myocardial perfusion maps at day 45 post‐MI in 2 pigs injected with vehicle (ID# 4146) or sAPCs (ID# 4126) (G) Perfusion maps were scaled between 20 and 250 mL/min per 100 g. *P<0.05 and **P<0.01 vs day 5). CMR indicates cardiac magnetic resonance; ID, identity number; LVEDV, left ventricular end diastolic volume; LVEF, left ventricular ejection fraction; LVESV, left ventricular end systolic volume; MI, myocardial infarction; sAPCs, swine adventitial pericytes.

Similar to the human study, CMR assessment of the heart structure and function showed no difference in groups receiving sAPCs or vehicle (Figure 9, Table 6 and Videos S5 through S8).

### Microvascular Outcomes of the Cell Therapy Studies

**Figure 10:**
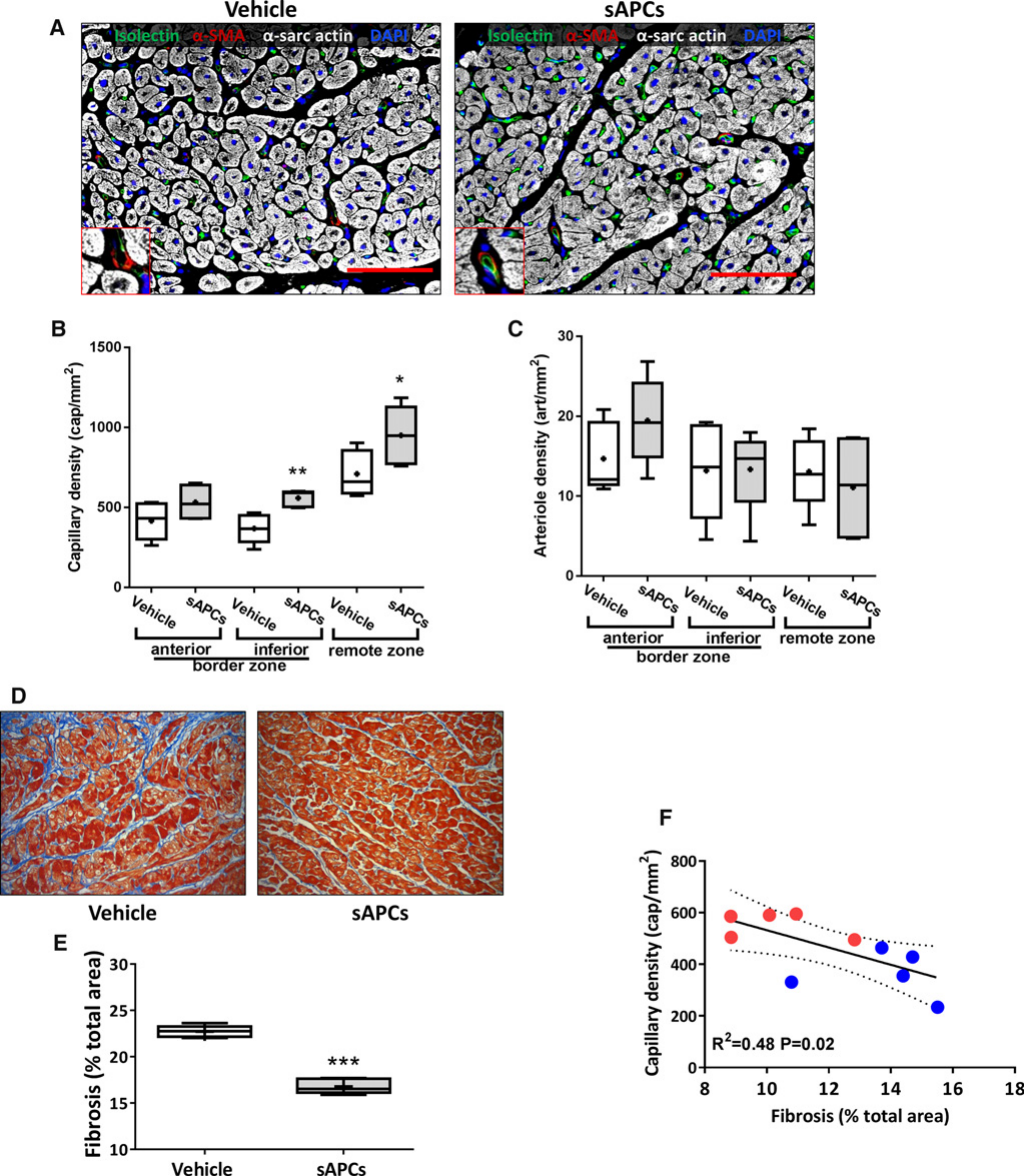
Benefit of sAPC therapy on microvascular density and fibrosis. A through C, Representative images (scale bar=100 μm) (A) and bar graphs showing the effect of cell therapy with sAPCs on capillary (B) and arteriole density (C). Representative images refers to hearts injected with vehicle or sAPCs (donor swine cell line ID# 5). The inserts illustrate arterioles stained with α‐SMA. Data are reported for 2 areas in the border zone and the remote zone. D, Illustrative microscopy images (total optical magnification ×200) and (E) bar graph illustrating the quantification of the fibrotic myocardium in the infarct border zone of swine hearts injected with vehicle or sAPCs (donor swine cell line ID# 2). *P<0.05, **P<0.01, ***P<0.001 vs vehicle. F, Correlation of capillary density and myocardial fibrosis in the whole population on swine injected with vehicle (blue dots) or sAPCs (red dots). α‐SMA indicates α‐smooth muscle actin; ID, identity number; sAPCs, swine adventitial pericytes.

General examination excluded the occurrence of malignant degeneration or calcification in hearts from the 2 treatment groups. We next investigated the effect of sAPC therapy on microvascular and fibrosis outcomes. The injection of sAPCs increased the capillary density in the inferior segment of the peri‐infarct zone (51%, *P*=0.003 versus vehicle) and the remote zone (34%, *P*=0.04 versus vehicle) (Figure 10A and 10B). Arteriole density was not affected in any of the segments examined (Figure 10C).

Morphometric analysis of myocardial fibrosis in the infarct border zone indicates a beneficial effect of sAPC therapy (−26% reduction versus vehicle, Figure 10D and 10E). The amount of myocardial fibrosis was inversely correlated with capillary density in the whole population of injected swine with vehicle or sAPCs (Figure 10F).

**Figure 11:**
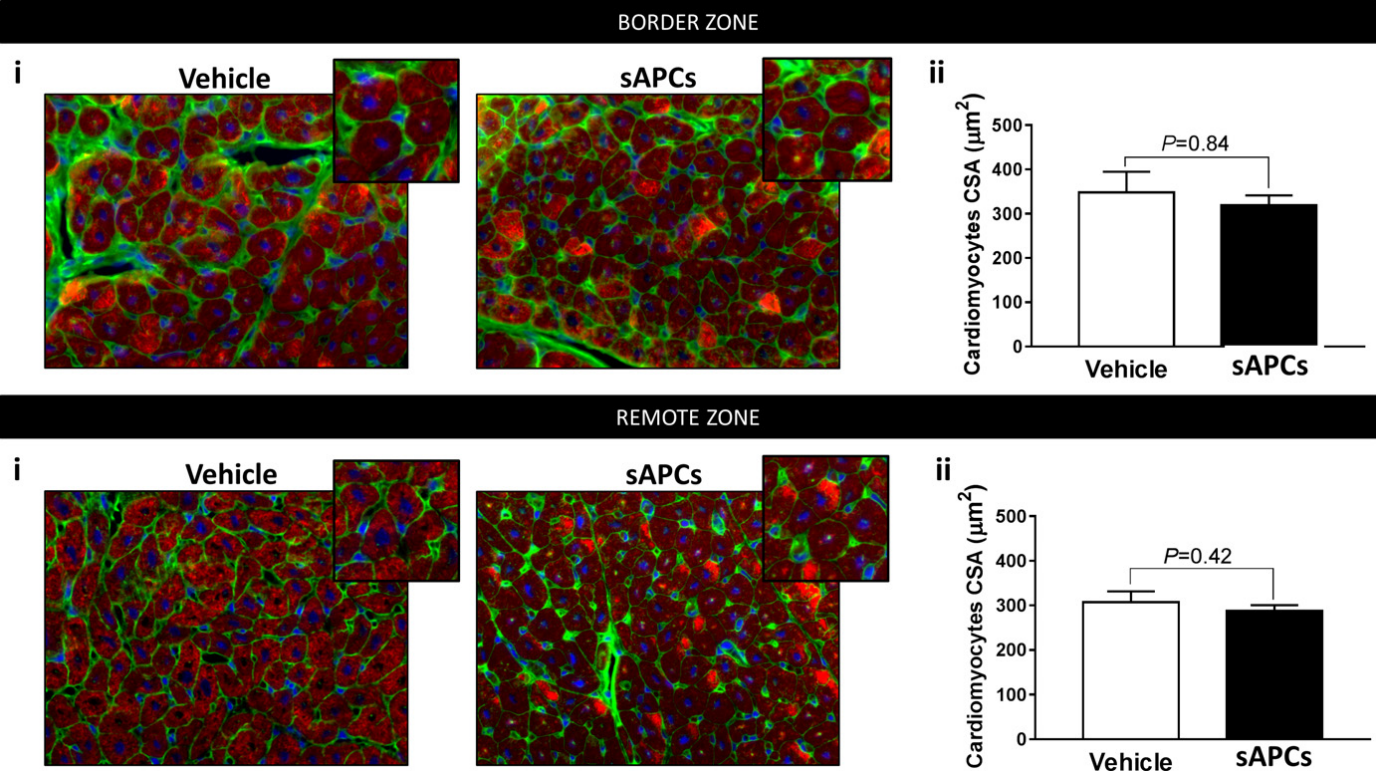
Morphometric evaluation of cardiomyocytes hypertrophy. A and B, CSA was measured in hearts of animals given vehicle or sAPC therapy. Ai and Bi: Representative immunofluorescence images of Wheat Germ Agglutinin (green), α‐Sarc Actin (red), and DAPI (blue) in the border and remote zones. Inserts show cell size at a higher magnification. Total optical magnification ×400. Aii and Bii: Histograms summarize quantitative data of CSA. Data are presented as mean±SEM. N=5 per each group. Difference between groups was measured by unpaired Mann–Whitney test, and *P* value reported on the graphs. α‐sarc actin indicates α‐sarcomeric actin; CSA, cell cross‐sectional area; DAPI, 4′,6‐diamidino‐2‐phenylindole; sAPCs, swine adventitial pericytes.

In addition, the assessment of cardiomyocyte remodeling by calculation of the myocyte cross‐sectional area denoted no difference between groups (Figure 11).

### Engraftment of sAPCs

**Figure 12:**
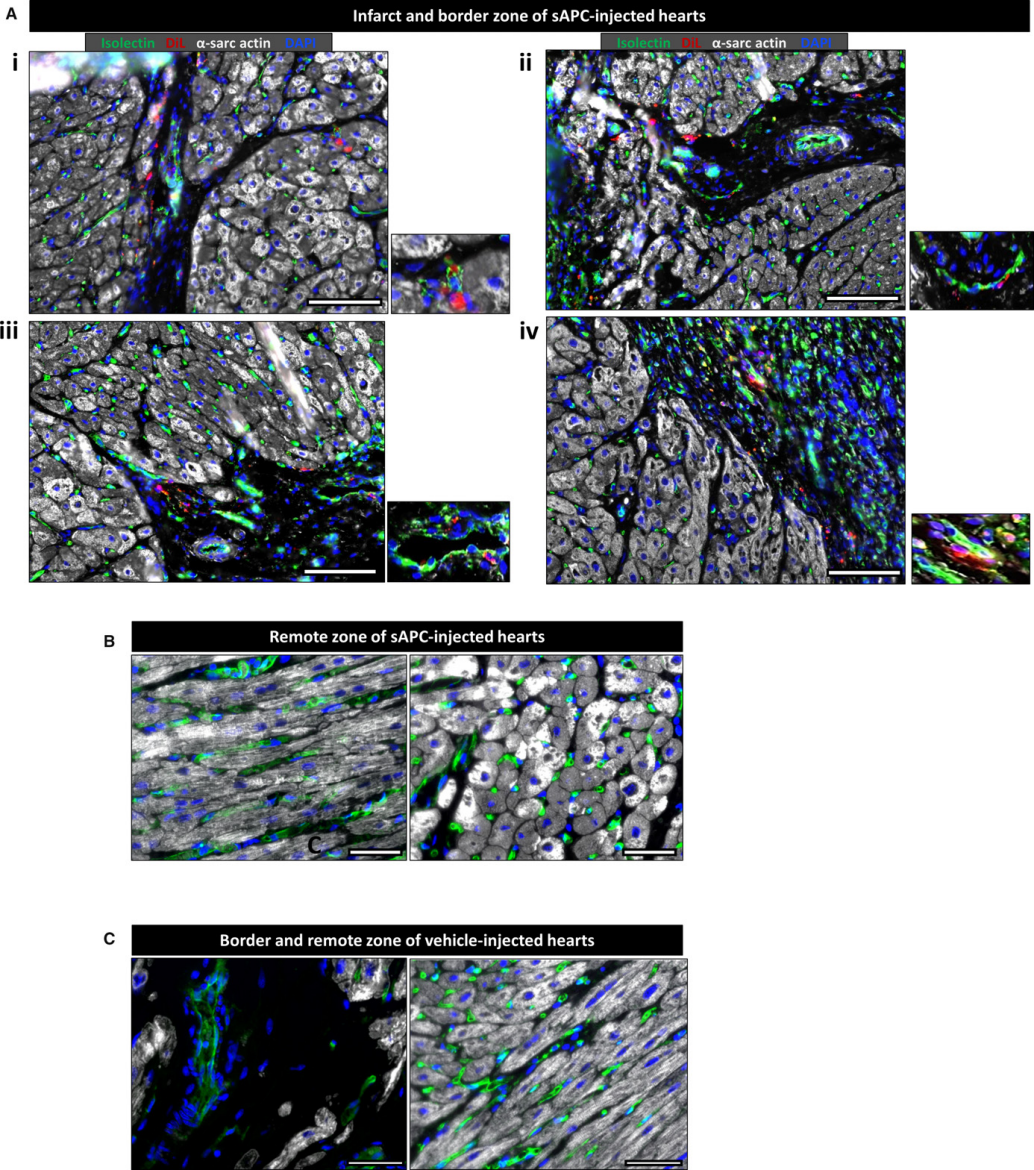
Engraftment of sAPCs in infarcted hearts. A, Representative immunofluorescence images showing engraftment of sAPCs in the infarct and nearby border zone (N=2). sAPCs are recognized by the red fluorescence of the Vybrant Dil marker, which stains cell membranes. In addition, α‐Sarc Actin (white) identifies cardiomyocytes. Isolectin B4 (green) has been used as a marker for endothelial cells, while DAPI (blue) shows nuclei. sAPCs can be seen at a higher magnification in the inserts placed on the right of each image. B, Representative images showing absence of sAPCs to the remote zone. C, Images taken from a vehicle‐injected heart as a negative control for the Vybrant Dil staining. For all images, total optical magnification is ×200. The scale bar in all images is 50 μm. α‐sarc actin indicates α‐sarcomeric actin; DAPI, 4′,6‐diamidino‐2‐phenylindole; Dil, 1,1′‐Dioctadecyl‐3,3,3′,3′‐Tetramethylindocarbocyanine; sAPCs, swine adventitial pericytes.

Two swine underwent the MI protocol and were injected 5 days later with sAPCs. The dose of cells injected in each swine is reported in Table 4. No casualties occurred in this experiment. Immunohistochemistry of hearts collected at 5 days posttransplantation indicates the presence of DiI‐labeled swine cells in the infarct and peri‐infarct border zone, with a few of them surrounding the isolectin‐positive vascular profiles and others dispersed in the scar (Figure 12A). No signal from injected swine cells was detected in the remote zone (Figure 12B). As expected, controls hearts injected with vehicle did not show any staining for the labeling tracer (Figure 12C). There was no evidence for the differentiation of injected cells into cardiomyocytes. The observed engraftment is in line with our previous study in mice, where Dil‐labeled hAPCs were found located in the infarct and infarct border zone.^16^

## Discussion

Mounting evidence from qualitative and quantitative research indicates that the triangulation of results from different models, sources, and methodologies can help achieve a deeper understanding of complex phenomena and synergize the trustworthiness and translatability of a scientific account.^29^, ^30^ Nevertheless, neither the US Food and Drug Administration nor the European Medicine Agency have yet provided clear guidelines on whether cell therapy should be tested in 1 or more animal species and, in the latter case, how the nature and importance of data from different models should be weighed before a clinical trial can begin. Furthermore, an interesting and yet unresolved controversy surrounds the preclinical choice of using human cells, which represent the final therapeutic product but carry the problem of being xenogeneic, or the corresponding animal products, which may better simulate the current allogeneic/autologous approach of clinical cell therapy.

In an attempt to resolve these fundamental issues, here, we report the first‐ever study of human and swine APC therapy in a large animal model of acute reperfused MI. The internal validity of our investigation was ensured at several key levels, with the randomized treatment groups being properly balanced for basal measurements, receiving identical care and assessment, and having similarly low levels of participant loss at final follow‐up. Furthermore, we used the ARRIVE consensus checklist to certify the proper and complete reporting of research data.^15^ Results obtained in swine were critically compared with previous studies conducted by us in mice (Table 7), to draw more definitive conclusions about the safety and efficacy of this new cell therapy product.

**Table 7:** Comparison of 3 Blind, Randomized, Placebo‐Controlled Studies Using APCs in Mice and Swine

Publication Reference (authors)	Katare et al^16^	Avolio et al^17^	Current study
Transplanted cell type	hAPCs	hAPCs	hAPCs and sAPCs
Human cell donor age, y	Not reported	57±6	73±3*
Human cell donor sex (M/F)	Not reported	8/1	6/1
Cell dose and route	hAPCs: 3×10^5^ I.M. hAPCs: 1×10^6^ I.M.	hAPCs:3×10^5^ I.M.	hAPCs: 21.4×10^6^ (range 16–28×10^6^ I.M. sAPCs: 10×10^6^ I.M.
Control for transplanted cells	Vehicle and hMSCs	Vehicle and hCSCs	Vehicle
Recipient species	Male immunodeficient CD1‐FOXOnu/nu mice and immunocompetent CD1 mice	Female immunodeficient SCID mice	Female immunocompetent Large‐White swine
Interventional MI	LAD permanent occlusion	LAD permanent occlusion	LAD artery temporary occlusion/reperfusion
Group size (n/treatment group)	14	5 to 7	hAPCs study: 8–9 sAPC study: 5–7
Loss of participants at final follow‐up	14 out of 28 (50%) Unbalanced between treatments (vehicle: 9; APCs: 5)	End points evaluated only at the final follow‐up assessment	hAPCs study: 1 out of 17 (5.8%)† sAPCs study: 2 out of 12 (16.7%)* Balanced (vehicle: 1; APCs: 2)
Outcomes			
Safety	Yes	Yes	Yes
LVEF (change vs vehicle)	+8% in FOXnu/nu mice at 14 d +15% in CD1 mice at 42 d	+29% in SCID mice at 14 d +12% in SCID mice at 42 d	Not significant
Effect on infarct size	Not determined	Morphometry: Not significant at 14 or 42 d	CMR: Not significant at 45 d
Effect on fibrosis in border zone	Yes	Yes	hAPCs: yes sAPCs: yes
Effect on capillary/arteriole density	Yes	Yes	hAPCs: no sAPCs: capillary density—yes sAPCs: arteriole density—no
Effect on perfusion	Yes	Yes	No
APC engraftment (d postinjection)	hAPCs detected at 5 d in border zone, at 14 d in infarct	hAPCs detected at 5 d in border zone	hAPCs not detected at 5 d sAPCs detected at 5 d in border‐zone

CMR indicates cardiac magnetic resonance; hAPCs, human adventitial pericytes; hCSCs, human cardiac stem cells; hMSCs, human mesenchymal stromal cells; I.M., intramuscular; LAD, left anterior descendent; LVEF, left ventricular ejection fraction; M/F, male/female; MI, myocardial infarction; sAPCs, swine adventitial pericytes.

*P<0.5 and ^†^ *P*<0.01 vs mouse study.

Genomic stability has a crucial role in cell‐based therapies. Aneuploidy was observed in in vitro‐cultured bone marrow‐derived mesenchymal stromal cells and adipose tissue–derived mesenchymal stromal cells during culture expansion, although cells with genomic alterations do not necessarily undergo malignant transformation.^31^, ^32^, ^33^ By using both traditional karyotyping and array‐based comparative genomic hybridization, we demonstrate that hAPC expansion does not result in significant mutations within the genome, thus providing novel reassurance on the safety of the cell product. On the other hand, in vitro cytotoxicity experiments and in vivo engraftment studies indicate rejection of hAPCs, because of xenogeneic antigen recognition by swine T cells. These new data contrast with the apparent tolerance of hAPCs by the murine immune system.^9^, ^16^ Although fundamental immunological mechanisms have strong similarities among mammalians, environmental and pathogen pressure have led immune systems of swine and mice to markedly diverge from each other, such that results obtained in 1 species are not always transferable to another.^34^ We observed a second layer of species incompatibility when performing coculture of sAPCs and human endothelial cells, which unexpectedly resulted in inhibition of in vitro network formation by the latter.

Because of the translational hurdles of the xenogeneic strategy, we successfully adopted the alternative strategy of obtaining pericytes from swine veins through a slightly modified version of the human isolation protocol. The swine cell product showed close similarities with hAPCs as assessed by immunocytochemistry, flow cytometry, and functional assays. This proxy cell product represents an invaluable material, considering the increasing popularity of swine as a translational research model and potential cell/organ donor.

Importantly, the in vitro cytotoxicity assays and in vivo engraftment studies showed that transplantation of allogeneic sAPCs in the swine heart is feasible and immunologically acceptable. Moreover, the safety of the proposed cell therapy product was confirmed by visual examination of animal conditions and behavior and measurement of body weight, hematocrit, and gross pathology of the heart. Quantification of cell engraftment by immunosorting, a method we successfully adopted in mice,^18^ is challenging in large animals such as swine, because of the difficulties in accurately retrieving a relatively low number of injected cells from the resident cell population, which is estimated to be in the range of 10 billion cells. Imaging analyses have the potential to provide semiquantitative information noninvasively, but available methods for assessing cell engraftment still have limitations.^35^ In the present study, the presence of transplanted sAPCs was confirmed by immunohistochemistry after 10 days postinfarction and 5 days from cell injection, whereas the clinical benefits were studied after 45 days. Therefore, it is not possible to know how long the transplanted cells persisted in the myocardium. These aspects represent a limitation of our investigation.

With regard to efficacy, the present study was consistent with our previous publications showing a benefit in microvascular angiogenesis and interstitial fibrosis.^16^, ^17^ Neovascularization and collagen deposition in the uninjured myocardium are important determinants of the healing response to an acute MI. Therefore, the ability of APCs to impact on these 2 parameters has considerable clinical value. When considering underpinning mechanisms, the present study recapitulates our previous results showing the APC inability to differentiate into endothelial cell or cardiomyocyte lineages.^18^ Paracrine mechanisms play a key role in the proangiogenic action of cell therapy,^36^ and we previously demonstrated the validity of this concept by gene silencing of angiogenic pathways in pericytes.^16^, ^37^ For instance, hAPCs express and release proangiogenic and antifibrotic factors, such as VEGF‐A, leptin, and miR‐132, with the secretion of both being increased after hypoxia.^9^, ^16^, ^37^ Western blot analysis showed that the VEGF‐A contained in the sAPC‐CM corresponds to the mature 22‐kDa protein. This was also accompanied by larger VEGF‐A bands. The antibody detection of these bands suggests that multiple molecules are bound together in a multimerized complex. Alternatively, VEGF‐A may be bound to another molecule, such as secreted VEGF receptors, with the region bound by the antibody exposed/separate from the region bound by the corresponding molecule/receptor. Both possibilities are supported by published evidence.^38^, ^39^

Swine and murine studies were also concordant in excluding an effect of APCs on the infarct size as determined by CMR and morphometry, respectively. On the other hand, the present swine study does not replicate the positive outcomes on cardiac function observed previously in mice.^16^, ^17^ Several factors may account for the discrepancy in functional outcomes. An important difference between the present investigation and studies in mice consists of the infarction model (temporary versus permanent left anterior descending occlusion). Furthermore, permanent left anterior descending occlusion resulted in lower LVEF values in mice^16^, ^17^ as compared with the reperfused swine model (≈20% versus 37%, respectively). The importance of baseline LVEF in influencing the outcome of cell therapy remains controversial. Two recent meta‐analyses of bone marrow cell therapy trials in patients with acute MI indicate that patients experienced similar improvement in LVEF regardless of the baseline LVEF. However, improvements in left ventricular end‐systolic volume were more pronounced in patients with lower baseline LVEF.^26^, ^40^ In contrast, in trials of chronic myocardial ischemia, the increase in LVEF elicited by cell therapy was significant only in the group with lower LVEF at baseline.^40^ Therefore, the lack of contractility benefit by APC therapy in swine may be related to the mild impact of reperfused MI on LVEF.

Another important difference of this large animal study consists of having assessed functional outcomes at 1 single follow‐up time point. Sequential and more prolonged evaluation of cardiac function might help to interpret the discrepancies between CRM and anatomical outcomes. For instance, myocardial perfusion as determined by CMR was not improved in the animals injected with sAPCs despite an increase of capillary density observed in histology. We believe this might be the consequence of several factors. Firstly, myocardial perfusion, as well as other in vivo functional and hemodynamic parameters, might be influenced by anesthesia with additional potential inter‐ and intraindividual variations in experimental models. Secondly, we did not perform pharmacological stress tests, which might help to enhance the sensitivity of the perfusion assessment. Thirdly, newly formed microvessels might need a longer time to achieve optimal functional capacity. An increase in capillaries rather than arterioles was observed by histology. Arterioles are well known to be mainly responsible for the regulation of tissue blood flow; therefore it might be the case that CMR was not able to detect subtle changes in myocardial perfusion. Finally, the beneficial effect of an enhanced capillary bed on cardiomyocyte survival may have been blunted by microvascular vasoconstriction. Recent investigation indicates that pericyte constriction contributes to coronary no‐reflow after postischemic reperfusion of the cardiac microcirculation.^41^ It would be of paramount importance to investigate whether APC transplantation impacts on postreperfusion microvascular constriction directly or through paracrine mechanisms and if, conversely, pharmacological control of coronary microvascular constriction may enhance the restorative action of APC therapy.

Another key factor to consider is the cell dose and, with regard to the hAPC transplantation study, the age of the cell donors. A meta‐analysis of 10 cell therapy studies in large animal models reports a median injected dose (12×10^6^ cells), which is similar to the doses used in our study.^8^ Our previous investigation in mice included a dose titration experiment showing that intramyocardial transplantation of 3×10^5^ hAPCs is sufficient to achieve significant therapeutic effects.^16^ In swine, we injected hAPCs and sAPCs at 53‐ to 93‐fold (16–28×10^6^) and 33‐fold (10×10^6^) higher concentrations, respectively. Since the swine heart is ≈1000 times heavier than the mouse heart, these doses might be therapeutically suboptimal. It should also be noted that despite injected doses of human cells being greater than the dose elected for swine cell studies, the latter exerted superior therapeutic effects. It is likely that the majority of the human cells were lost because of the immune response of the recipient. Additionally, they may have caused other inadvertent adverse phenomena attributable to their xenogeneic nature.

Aging causes a decline in the function and regenerative capacity of human cells.^42^ Human donors of the present swine study were older than those recruited in the mouse study (73±3 years versus 57±6 years, respectively).^17^ Therefore, the advanced age of the donors, in addition to the host reaction to these cells, might have contributed to the failure of hAPCs to exert the benefits seen in murine studies^12^, ^13^ as well as in swine studies where allogeneic sAPCs were sourced from young donor animals.

## Conclusion

In summary, the results from this preclinical study support the feasibility and safety of intramyocardially injected APCs for the treatment of MI. The impact on efficacy appears confined to an improvement of vascularization and reduction of fibrosis, which was still not enough to improve function and contractile indices. When comparing this finding with the previous observation that contractility is improved in mice with nonreperfused acute MI,^16^ we speculate that APC therapy might be especially amenable to patients with nonrevascularizable coronary artery disease. The majority of these patients have refractory angina, which is unresponsive to optimal treatment and associated with a markedly impaired quality of life.

Our proof of concept study was conducted to assess the safety and therapeutic value of pericytes as a monotherapy. It would be of paramount importance to test pericytes in combination with optimal drug therapy for MI. Another attractive possibility is the association with other dedicated approaches of regenerative medicine. Conjoint cardiomyogenic treatments, for instance, using combinatory transplantation of cardiac stem cells as shown by us previously^17^—exosomes and vesicles secreted by cardiac stem cells^43^, ^44^ or exogenous administration of selected mitosis‐related genes or microRNAs^45^, ^46^, ^47^, ^48^—may result in synergistic benefit with the restoration of microvascular perfusion and contractile function.

Finally, triangulation with data from our studies in mice provides an invaluable method to interpret the experimental results, but also calls for more standard reporting guidelines for animal studies in cardiac regeneration.

## Author Contributions

Alvino and Rodriguez‐Arabaloaza performed the in vitro and histology studies and prepared cells for transplantation. Slater contributed to expressional studies. Mangialardi contributed to cytotoxic experiments. Culliford provided expert opinion in the pathway to clinical translation. Avolio and Alvino contributed to the characterization of human and swine cells. Spencer performed the study of cell incorporation. Hassan performed additional experiments on VEGF and TBX18 expression. Sueiro Ballesteros has overseen the use of the Novocyte machine and FlowJo software in flow cytometry studies. Hennessey and Delmege contributed to studies of genetic stability. Angelini provided clinical interpretation of the data. Ascione, Emanueli, and Angelini secured funding and participated in designing the study and providing the clinical interpretation of results. Fernández‐Jiménez and Galán‐Arriola performed the closed chest MI studies in swine. Ayaon‐Albarrán performed the cardiac surgeries and did the blinded intramyocardial injection of APCs or vehicle. Sánchez‐González designed and performed the CMR protocols and examinations. Fernández‐Jiménez and Galán‐Arriola did all CMR analyses blind to treatment allocation. Ibanez designed and supervised all swine in vivo studies, interpreted CMR results, and is fully responsible for all in vivo swine studies and CMR data. Madeddu secured funding, conceived the study, and wrote the article.

## Sources of Funding

Madeddu is the recipient of grants from the British Heart Foundation and the Medical Research Council in support of research on human and swine APCs. Angelini is the recipient of a grant from the NIHR Biomedical Centre at the University Hospitals Bristol NHS Foundation Trust, which partially supported the present study. Fernández‐Jiménez was the recipient of nonoverlapping grants from the Ministry of Economy, Industry, and Competitiveness through the Instituto de Salud Carlos III (Rio Hortega fellowship); and the Fundación Jesús Serra, the Fundación Interhospitalaria de Investigación Cardiovascular (FIC), and the CNIC (FICNIC fellowship). The use of QMass software was partly supported by a scientific collaboration between the CNIC and Medis Medical Imaging Systems BV. This study forms part of a Master Research Agreement (MRA) between the CNIC and Philips Healthcare. The CNIC is supported by the Ministry of Economy, Industry and Competitiveness (MINECO) and the Pro‐CNIC Foundation, and is a Severo Ochoa Center of Excellence (MINECO award SEV‐2015‐0505).

## Disclosures

Sánchez‐González is a Philips Healthcare employee. The other authors declare no conflict of interest.

## Supporting information


**Data S1.** Extended Materials and Methods.
**Table S1.** Antibodies Used in Immunocytochemistry Studies of sAPCs
**Table S2.** Antibodies Used in Flow Cytometry Studies on sAPCs
**Table S3.** *Taqman* Probes Used in the Molecular Biology Studies of sAPCs
**Figure S1.** Genetic analysis of expanded hAPCs.
**Figure S2.** Mesenchymal differentiation of sAPCs.
**Figure S3.** Cytotoxic activity of sAPCs and swine splenocytes in suspension.
**Videos S1 and S2.** Vehicle‐injected swine (ID# 2644) with CMR imaging captured at 5 d (1) or 45 d post‐MI (2). Format: Windows Media Video file (WMV).
**Videos S3 and S4.** hAPC‐injected swine (ID#2647) with CMR imaging captured at 5 d (3) or 45 d post‐MI (4). Format: Windows Media Video file (WMV).
**Videos S5 and S6.** Vehicle‐injected swine (ID#4146) with CMR imaging captured at 5 d (5) or 45 d post‐MI (6). Format: Windows Media Video file (WMV).
**Videos S7 and S8.** sAPC‐injected swine (ID#4126) with CMR imaging captured at 5 d (7) or 45 d post‐MI (8). Format: Windows Media Video file (WMV).
