# Supplementary material for: Transplantation of Allogeneic Pericytes Improves Myocardial Vascularization and Reduces Interstitial Fibrosis in a Swine Model of Reperfused Acute Myocardial Infarction
Source: J Am Heart Assoc. 2018 Jan 22;7(2):e006727. doi: 10.1161/JAHA.117.006727 (PMC5850145; doi:10.1161/JAHA.117.006727)
Supplement: Supplementary file 1 — Data S1. Extended Materials and Methods. Table S1. Antibodies Used in Immunocytochemistry Studies of sAPCs Table S2. Antibodies Used in Flow Cytometry Studies on sAPCs Table S3. Taqman Probes Used in the Molecular Biology Studies of sAPCs Figure S1. Genetic analysis of expanded hAPCs. Figure S2. Mesenchymal differentiation of sAPCs. Figure S3. Cytotoxic activity of sAPCs and swine splenocytes in suspension. Videos S1 and S2. Vehicle‐injected swine (ID# 2644) with CMR imaging captured at 5 d (1) or 45 d post‐MI (2). Format: Windows Media Video file (WMV). Videos S3 and S4. hAPC‐injected swine (ID#2647) with CMR imaging captured at 5 d (3) or 45 d post‐MI (4). Format: Windows Media Video file (WMV). Videos S5 and S6. Vehicle‐injected swine (ID#4146) with CMR imaging captured at 5 d (5) or 45 d post‐MI (6). Format: Windows Media Video file (WMV). Videos S7 and S8. sAPC‐injected swine (ID#4126) with CMR imaging captured at 5 d (7) or 45 d post‐MI (8). Format: Windows Media Video file (WMV). [file JAH3-7-e006727-s001.pdf]

SUPPLEMENTAL MATERIAL

Data S1.

Extended Materials and Methods

Assessment of hAPC genetic stability

The following whole genome analysis testing strategy was carried out on five cell lines and the source vein tissue:

(i) Chromosome analysis of G-banded metaphases from the earliest possible passage (P2) and P7 cell cultures. This test detects both balanced and unbalanced chromosome rearrangements. Briefly, cultured cells were harvested using a robotic liquid handling system from Genial Genetic Solutions (Multiprep Genie 205), seeded on coverslips and fixed in Carnoy's fixative. Metaphase chromosomes were G-banded using Trypsin and Leishman stain for karyotype analysis. Images of selected cells were captured using the MetaSystems cell finding and image analysis equipment and then karyotyped. Chromosome analysis was carried out to a quality standard QA4. According to the number of available metaphases, studies were performed on 10 or 20 cells.

(ii) Microarray comparative genomic hybridization (CGH) of DNA extracted from vein tissue against DNA extracted from P7 cultured hAPCs. Pooled, sex-matched DNA was used as a control. GGH analysis detects acquired copy number changes present in a large proportion of cells. The assay was performed using the OGT 8x60K ISCA v2.0 array (Oxford Gene Technology, UK), which has an average resolution of 180kb. Data were analyzed with OGT CytoSure Interpret v4.7.

APC immunogenic activity mediated by natural antibodies and complement

Studies were carried out to verify the possibility that hAPCs trigger an immune response mediated by pre-existing xenoreactive antibodies and complement activation, which may contribute to immune rejection following transplantation in swine.

We first determined if porcine serum contains natural antibodies that can recognize human antigens. To this aim, hAPCs were grown to confluence in a 96-well plate, fixed with 0.1% glutaraldehyde for 5min at +4° C and then blocked with PBS supplemented with BSA 1% for 30min. After washing, cells were incubated for 1h at +37° C with undiluted or progressively diluted pre-immune porcine serum (Innovative Research, IPG-COMPL) or human serum as a negative control. Cells were washed three times with PBS and anti-porcine IgG or anti-porcine IgM were added to the wells for 2h at +37°C. After washing, p-nitrophenylphosphate (Sigma-Aldrich, AP0100) in diethanolamine was added to the wells as a substrate and after 5min plates were read at 405 nm.

To assess complement activation, hAPCs were incubated with undiluted or serial dilutions of porcine pre-immune serum or human serum as a negative control for 1h at +37°C followed by incubation with complemented porcine serum for 1h at +37°C. The cells were washed three times with PBS, fixed with 0.1% glutaraldehyde for 5min at +4° C and then blocked with PBS supplemented with BSA 1% for 30min, washed twice again with PBS, and then blocked with 1% BSA. Complement activation was determined by measuring the deposition of C3b by ELISA (Antibodies-online, ABIN1560930), according to manufacturer's instructions.

APC immunogenic activity mediated by antigen-specific cytotoxic T-lymphocytes

Isolation of effector cells

Additional studies were performed to evaluate the possibility that xenogenic hAPCs or allogenic sAPCs may trigger an immune response by antigen-specific cytotoxic T-lymphocytes. To obtain effector cells, spleens were harvested from swine, minced in Petri dishes and crushed through a 70µm cell strainer with the help of a 2mL syringe plunger. Strainers were washed five times with 1x PBS (Life Technologies, 14190250) to release the splenocytes into the Petri dishes. The cell suspension was transferred into 50mL tubes containing 15mL of Histopaque-1077 (Sigma-Aldrich, 10771) to separate the mononuclear fraction by gradient centrifugation (400xg for 30min at RT with no accelerator/brake). Cells were transferred into a new 50mL tube, washed with 30mL of 1x PBS and centrifuged at 400xg for 10min at RT. For erythrocytes exclusion, the resulting cell

pellet was resuspended in 1mL of 1x PBS and incubated in 5mL of 1x Red Blood Cells lysis buffer (BioLegend, 420301) for 10min at RT and protected from light. Then, cells were washed with 30mL of 1x PBS and centrifuged at 400xg for 10min at RT. Live cells counting was performed using trypan blue exclusion. Then, $1x10^6$ cells/mL were seeded in RPMI 1640 plus GlutaMAX medium (Life Technologies, 61870010) supplemented with 10% (v/v) heat-inactivated Fetal Bovine Serum (FBS) (Life Technologies, 10500064) 100 IU/mL penicillin and 0.1 mg/mL streptomycin (Life Technologies, 15070063). To stimulate Natural Killer lymphocytes (NK) as effector cells for the cytotoxic assay, the culture media was enriched with 100 IU/mL recombinant porcine interleukin-2 (R&D Systems, 652-P2) and 25 ng/mL recombinant porcine interleukin -12 (R&D Systems, 912-PL) for 48h before the assay.

Cytotoxic assay and flow cytometry analysis

For the cytotoxic assay, splenocytes were challenged with hAPCs or sAPCs. Briefly, $5x10^5$ target cells (hAPCs or sAPCs, three biological replicates) were seeded in 12 multi-well plates together with stimulated effector cells at four different target cell/effector cell ratios: 1:0, 1:1.5, 1:3 and 1:6. Target and effector cells were co-cultured in a humidified 5% CO2 incubator at +37°C for 4h in EGM-2. In a separate experiment, $3x10^5$ sAPCs were seeded in 6 multi-well plates and let them attach for 48h. Then, stimulated effector cells were added to have the same ratio as above. Effector and target cells were co-cultured in a humidified 5% CO2 incubator at +37°C in EGM-2 for 4h.

For antibody staining, cell suspensions or trypsinized cells were transferred into 5mL polystyrene tubes (Corning, 352054) and centrifuged at 400xg for 10min at +4°C. Cell pellets were resuspended in 100 µL of FACS buffer (1% BSA (v/v) + 2mM EDTA in 1x PBS) and incubated with the early apoptotic marker FITC Annexin V (1:50, BD Pharmingen, 556419) for 30min at +4°C. Propidium iodide (1:400, Invitrogen, P3566) was then added to cells. At least $1x10^4$ cells were acquired per sample with the same settings. Early and late apoptotic target cells were assessed measuring the percentage of Annexin V+/P.I.- and Annexin V+/P.I.+ respectively, using FlowJo software (FlowJo, LLD). Necrotic cells were identified as Annexin V-/P.I.+.The gating strategy included a step to distinguish effector and target cells using the major human histocompatibility antigen HLA (BioLegend, 311410) or labelling effector cells with a Violet Cell Tracer (Invitrogen, C34557).

Flow cytometry

To optimize the flow cytometry analysis and exclude the occurrence of hematopoietic cell contamination, we adopted a single-step staining - Fluorescence Minus One (FMO) protocol on three APC lines (the ones used for *in vivo* transplantation in the swine MI model, *vide infra*) and control swine MNCs. The FMO methodology is recommended in multiple fluorescence studies, like the one used here, to ensure that any spread of the fluorochromes into the channel of interest is properly identified. In addition, we used the Fixable Viability Dye eFluor 780 to label dead cells and exclude them from the gating strategy. The size of hAPCs and sAPCs was calculated using the Tali™ Image-based cytometer (Invitrogen) and Novocyte 3000 (Acea Biosciences). A total of $2x10^5$ cells were incubated with swine and human mesenchymal antibody, CD105 (LifeSpan), conjugated with phycoerythrin (PE), a bright and well-excited fluorophore once the laser light source passes through the cells. Also, CD105-PE ensures more specificity to the cell size measurement. Once stained, the cells were fixed with 1% (v/v) PFA in PBS. Unfixed and unstained cells were used to compare any cell size variation with fixed and stained cells.

Semi-quantitative PCR on sAPCs

Extracted total RNA (N=3 biological replicates) was reverse-transcribed into single-stranded cDNA using a High Capacity RNA-to-cDNA Kit (Life Technologies). The reverse transcription–PCR was performed using the first-strand cDNA with TaqMan Fast Universal PCR Master Mix (Life Technologies). Quantitative PCR was performed on a Quant Studio 6 Flex Real-Time PCR system (Applied Biosystems). PCR parameters for cycling were as follows: +50°C incubation for 2min, +95°C for 10min, 40 cycles of PCR at +95°C for 15sec, and +60°C for 1min. The following

Taqman assays were used: β-Actin (Ss033376160_u1), U6 (Ubiquitin 6, 001973), and PPIA (Peptidyl-prolyl isomerase A Ss03394782_g1) as a housekeepers; *for characterization of angiogenesis-related mRNAs and micro-RNAs:* VEGF-A (Ss03393990_m1), TBX18 (Hs01385457_m1), miR210 (3p CUGUGCGUGUGACAGCGGCUGA), and miR132 (3p UAACAGUCUACAGCCAUGGUCG); *for lineage characterization and purity assessment*: CD31 (Ss03392600_u1), CD45 (Ss03376444_u1), CD11b (Ss03374590_m1).

Differentiation of sAPCs

For induction of osteogenesis, sAPCs were cultured in Dulbecco's Modified Eagle medium (DMEM, Life Technology, UK) supplemented with L-ascorbic acid 2-phosphate (50µM), dexamethasone (0.1µM) and β-glycerol phosphate (10mM). For adipogenic differentiation cells were cultured in DMEM supplemented with dexamethasone (1µM), indomethacin (100µM), insulin (10µg/mL) and 3-isobutyl-1-methylxanthine (500µM). The media were replaced every three days.

Network formation

Swine or human APCs were stained with the long-term cell tracker VyBrant diL (Life Technologies, UK) and co-cultured with swine pulmonary artery endothelial cells (sPAECs) at a ratio 1:4 on Matrigel substrate (BD Biosciences, UK), containing Endothelial cell growth medium (EGM-2) and incubated for 6 hours. Each cell type was also seeded alone as control. In addition, to assess the effect of the secretome of the cells. sAPC and hAPC conditioned media (CM) diluted 1:2 with fresh EGM-2 and swine serum, were incubated with sPAECs on Matrigel for 6 hours.

Myocardial infarction procedure

The MI protocol is detailed elsewhere.[1, 2] Anesthesia was induced by intramuscular injection of ketamine (20 mg/kg), xylazine (2 mg/kg), and midazolam (0.5 mg/kg), and maintained by continuous intravenous infusion of ketamine (2 mg/kg/h), xylazine (0.2 mg/kg/h), and midazolam (0.2 mg/kg/h). Animals were intubated and mechanically ventilated with oxygen (fraction of inspired O_2: 28%). Central venous and arterial lines were inserted, and a single bolus of unfractioned heparin (300 IU/kg) was administered at the onset of instrumentation. The left anterior descending coronary artery, immediately distal to the origin of the first diagonal branch, was occluded for 50min with an angioplasty balloon introduced via the percutaneous femoral route using the Seldinger technique. Balloon location and maintenance of inflation were monitored angiographically. After balloon deflation, a coronary angiogram was recorded to confirm patency of the coronary artery. A continuous infusion of amiodarone (300 mg/h) was maintained during the procedure in all swine to prevent malignant ventricular arrhythmias. In cases of ventricular fibrillation, a biphasic defibrillator was used to deliver non-synchronized shocks.

CMR protocol

Baseline CMR scans were performed at day 5 post-MI immediately before randomization to vehicle or APC intra-myocardial injection, and subsequent follow-up CMR scans were performed at day 45 post-MI. All CMR examinations were conducted with a Philips 3-Tesla Achieva Tx whole body scanner (Philips Healthcare, Best, the Netherlands) equipped with a 32-element phased-array cardiac coil. The imaging protocol included The protocol included a standard segmented cine steady-state free-precession (SSFP) sequence to provide high-quality anatomical references, and to determine left-ventricle end-diastolic wall thickness, end-diastolic volume (LVEDV), end-systolic volume (LVESV) and left ventricular ejection fraction (LVEF);[3] a dynamic acquisition with dual-saturation technique during gadolinium-based contrast administration to determine absolute myocardial perfusion;[4] and a late gadolinium-enhanced (LGE) sequence to assess infarct size.[3]

All sequences were acquired in free-breathing mode. The imaging parameters for the SSFP sequence were FOV 280 x 280 mm, slice thickness 6 mm with no gap, TR 2.8 ms, TE 1.4 ms, flip angle 45°, cardiac phases 30, voxel size 1.8 x 1.8 mm, and 3 NEX. The first-pass perfusion was acquired by dynamic dual saturation-recovery gradient echo in 3 short-axis slices at the basal, mid-ventricular and apical level (TS=100ms). An extra slice was interleaved at the level of aorta output track to measure the vascular input function in the coronary with a shorter saturation

time (TS=20ms). All other parameters were equivalents between both acquisitions (slice thickness 6, gap 8, TR/TE: 2.6/1.3, Flip angle: 15°, FOV 280x300, Bandwidth: 460, SENSE factor =2). Before contras injection T1 mapping MOLLI sequence was acquired at the same levels to improve the accuracy to convert from signal intensity to contrast concentration. All animals received an intravenous dose of 0.1 mmol/Kg of gadolinium at 3 mL/s for first-pass perfusion imaging and an additional dose of 0.1 mmol/Kg of gadolinium at 3 mL/s for LGE imaging. Thus, LGE imaging was performed 10 to 15min after intravenous administration of 0.20 mmol of gadopentetate dimeglumine contrast agent per kg of body weight using a T1 inversion-recovery spoiled turbo field echo (T1-IR-TFE) sequence with the following parameters: FOV 280 × 280 mm, voxel size 1.6 × 1.6 mm, end-diastolic acquisition, thickness 6 mm with no gap, TR 5.6 ms, TE 2.8 ms, inversion delay time optimized to null normal myocardium, and 2 NEX.

SSFP and T1-IR-TFE sequences were performed to acquire 13 to 15 contiguous short-axis slices covering the heart from the base to the apex, whereas perfusion imaging was analyzed in a mid-apical ventricular short axis slice.

CMR analysis

CMR images were analyzed using dedicated software (MR Extended Work Space 2.6, Philips Healthcare, The Netherlands; and QMassMR 7.6, Medis, Leiden, The Netherlands) by two observers experienced in CMR analysis and blinded to treatment allocation. LV mass and ejection fraction, myocardial perfusion and extent of necrosis were determined.

LV cardiac borders were automatically traced with manual adjustment in each cine image to assess the LVEDV, LVESV, and LVEF. In the tracing convention used, the papillary muscles were included as part of the LV cavity volume. Ejection fraction (EF) was computed as EF= (LVEDV-LVESV)/LVEDV. LV epicardial borders were also traced on the end-diastolic images, with LV mass computed as the end-diastolic myocardial volume (i.e., the difference between the epicardial and endocardial volumes) multiplied by myocardial density (1.05 g/mL). Values of LV mass normalized to body surface area were calculated with modified Brody's formula.[5]

Before perfusion analysis, time intensity curves were transformed to contrast concentration using T1 values information. After contrast conversion, absolute flow values were obtained by pharmacokinetic signal modelling using the vascular input function and the swine hematocrit as correction factors. Myocardial perfusion expressed as mL/100g/min. On the absolute flow map regions of interest (ROI) of 0.5 cm^2 were manually drawn in a mid-ventricular slice at the core infarcted area, infarct borders and at the remote myocardium with special care not to include a ventricular cavity.

Myocardial necrosis (infarct size, IS), expressed as a percentage of LV mass, was defined according to the extent of late gadolinium enhancement after manually tracing the endocardial and epicardial contours on T1-IR-TFE short axis images. Abnormal areas were defined using the full-width at half-maximum, with manual correction if needed. Hypo-intense black areas within the necrotic zone, corresponding to microvascular obstruction, were included within the necrotic area.[6, 7]

Intramyocardial injection of vehicle and APCs

Injections in the peri-infarct zones of the LV was carried out *via* a left anterior mini-thoracotomy under general anaesthesia by an expert cardiac surgeon blind to the treatment allocation. Sedation was maintained with sevoflurane. Continuous intravenous infusion of fentanyl served as an analgesic during surgery, and a single dose of prophylactic antibiotic with cefuroxime was administered just before the procedure. Mechanical endotracheal ventilation was controlled by an external respirator.

A minimally invasive right lateral thoracotomy was then performed in the third intercostal space as previously described.[8, 9] Then, left internal mammary artery was dissected and ligated for a better visualization of peri-infarcted areas. Through a small peri-cardiotomy, the anterior and lateral LV walls were exposed, and a total of N=10 micro-injections (for a total volume ~3mL) were performed at the peri-infarct zones under direct vision. After administration was finished, the small

peri-cardiotomy was left open, the chest wall and skin incisions were closed and the pneumothorax was evacuated with a chest tube. Animals were extubated and sent to the recovery room. After surgery and before the animals recovered from anesthesia, intramuscular (0.01 mg/kg) buprenorphine was administered as a postoperative analgesia. During their recuperation, animals were cared for by dedicated veterinarians and technicians at the CNIC. All animals were returned to the farm before final follow-up CMR and sacrifice.

Collection and analyses of swine hearts

At sacrifice, animals were subjected to an overdose of anesthetic (ketamine, xylazine and midazolam) to collect hearts and perform histological analyses.

Samples processing for histology

After explantation of the porcine hearts, 4 different areas of the LV (Remote= REM, Peri-Infarct Anterior= PIA, Peri-Infarct Inferior= PII and Infarct= INF) were collected and washed once in 1x PBS prior to fixation with freshly prepared 4% (w/v) PFA (Sigma-Aldrich, 441244) in PBS for 48 hours at 4°C. After the fixation, heart samples were washed once with 1x PBS and included in paraffin and sectioned at a thickness of 5 µm for subsequent immunohistochemistry analysis.

Vascular density

For capillary density, paraffin sections were incubated with biotinylated Isolectin B4 diluted 1:200 (Life Technologies, I21414) overnight in a humidified chamber and at +4°C, followed by the incubation of streptavidin Alexa Fluor 488 diluted 1:200 (Life Technologies, S11223) 1h at RT. For arteriole density, the same sections were probed with α-Smooth Muscle Actin (α-SMA) antibody conjugated with Cy3 diluted 1:400 (Sigma-Aldrich, C6198) 1h at RT. Capillaries and arterioles were calculated in 40 fields (at X200 magnification) in the Peri-Infarct myocardium, and the final data expressed as the number of capillaries or arterioles per square millimeter. The analysis was performed using the free software ImageJ (http://imagej.nih.gov/ij/). Adobe Photoshop software was utilized to compose and overlay the images (Adobe).

Interstitial Fibrosis

Peri-Infarct paraffin sections were stained with Masson's trichrome stain for cardiac fibrosis quantification. Then, 20 pictures at X200 magnification were taken from each section for the subsequent fibrosis analysis with Image J software. The final data was expressed as the percentage of the fibrotic area from the total quantified area.

Table S1. Antibodies used in immunocytochemistry studies of sAPCs

Marker	Technique	Permeabilization	Reactivity	Primary antibody source and dilution	Secondary antibody source
NG2	ICC	Yes	Swine	NovusBiological, 1:50	Invitrogen, A488 Goat α-Rabbit
PDGFRβ	ICC	Yes	Swine	GeneTex, 1:100	Invitrogen, A488 Goat α-Mouse
CD34	ICC	Yes	Swine	GeneTex, 1:50	Invitrogen, A488 Goat α-Mouse
Vimentin	ICC	Yes	Human	Abcam, 1:400	Invitrogen, A488 Goat α-Rabbit
SOX2	ICC	Yes	Human/Swine	Merck Millipore, 1:100	Invitrogen, A488 Goat α-Rabbit
GATA-4	ICC	Yes	Human	Abcam, 1:100	Invitrogen, A488 Goat α-rabbit
OCT-4	ICC	Yes	Human/Swine	Abcam, 1:100	Invitrogen, A488 Goat α-Rabbit
CD31	ICC	Yes	Swine	Abcam, 1:20	Invitrogen, A488 Goat α-Rabbit
VE-cadherin	ICC	Yes	Human/Swine	Santa Cruz, 1:50	Invitrogen, A488 Goat α-Mouse
CD146	ICC	Yes	Human/Swine	Abcam, 1:100	Invitrogen, A488 Goat α-Rabbit
Biotinylated Isolectin-B4	IHC	Yes	Human/Swine	Invitrogen; 1:200	Invitrogen, A488 Streptavidin
α-SMA-Cy3	IHC	Yes	Human/Swine	Invitrogen; 1:100	
WGA-A488	IHC	Yes	Human/Swine	Invitrogen; 1:100	
α-SA	IHC	Yes	Human/Swine	Sigma; 1:200	Invitrogen, 649 Donkey-α mouse IgM
VEGF-A	WB	N/A	Human/Swine	Antibodies-Online; 1:200	GE Healthcare; ECL Donkey α-Rabbit IgG-HRP, 1:5000
TBX18	WB	N/A	Human	Santa Cruz; 1:400	GE Healthcare; ECL Sheep α-Mouse IgG-HRP, 1:5000
β-actin	WB	N/A	Human/Swine	Sigma; 1:30000	GE/Healthcare; ECL Sheep α-Mouse IgG-HRP, 1:5000

Cells (N=4 biological replicates run in triplicate) were fixed with freshly prepared 4% (W/V) PFA in PBS for 10min at +4°C , washed with PBS and probed with the indicated antibodies. For detection of intracellular antigens, cells were permeabilized for 10min at +4°C with 0.3% (v/v) Triton X-100 (Sigma-Aldrich) diluted in PBS. Cells were incubated with the indicated primary antibodies (16h at +4°C) and appropriate secondary antibodies (1:200 anti-rabbit Alexa 488 or 1:200 anti-mouse Alexa 488, 1h at +20°C in the dark). The nuclei were counterstained with DAPI (Sigma-Aldrich). Slides were mounted using Fluoromount-G (Sigma-Aldrich). Cells were analyzed at a 200X and 400X magnification. The PhotoPlus X6 software was utilized to compose and overlay the images (Adobe). Swine Pulmonary Artery Endothelial Cells (sPAECs, AMSBio, USA) at P4 were used as positive controls.

Table S2. Antibodies used in flow cytometry studies on sAPCs

Marker	Permeabilization	Reactivity	Primary antibody source dilution	Fluorophores
**CD90**	No	Swine	eBioscience, 1:20	Pe-Cy7
**CD44**	No	Swine	eBioscience, 1:20	APC
**CD105**	No	Swine	LifeSpan, 1:5	PE
**CD45**	No	Swine	Serotec, 1:25	FITC
**CD11b**	No	Human/Swine	eBioscience, 1:20	Pe-Cy7
**CD31**	No	Swine	Serotec, 1:80	PE
**CD146**	No	Swine	Serotec, 1:60	FITC
**PDGFRβ**	No	Human	Biolegend, 1:25	PE
**Dye eFluor 780**	No		eBioscience, 1:1000	APC-Cy7

Cells ($1x10^6$, N=3 biological replicates) were washed in 1X DPBS (Life Technologies) and treated with 10X trypsin/EDTA (Life Technologies). Then, cells were washed, spun at 400xg for 10min at 4°C and re-suspended in FACS-staining buffer containing 0.1% (v/v) BSA, 1mM EDTA and 0.1% (W/V) sodium azide, followed by blocking of unspecific binding with 10% (v/v) BSA in 1X DPBS. $2x10^5$ cells/tube were incubated with mesenchymal and hematopoietic antibodies for 30min, at +4°C, in the dark). For endothelial marker CD31, cells were incubated for 15min, on ice in the dark, followed by three washes in 1X DPBS. Fixable Viability Dye eFluor 780 (ebioscience, UK) was used for the exclusion of dead cells from the gating procedure. A single fixation with 1% (W/V) PFA was applied after the staining instead of a double fixation protocol routinely used in the hAPC-SOP. Swine PB-MNCs and PAECs were employed as a positive control for hematopoietic and endothelial markers, respectively.

Table S3. *Taqman* probes used in the molecular biology studies of sAPCs.

Gene	Species	Assay ID
**PPIA (housekeeper)**	Swine	Ss03394782_g1
**β-Actin (housekeeper)**	Swine	Ss033376160_u1
**UBC (housekeeper)**	Human	Hs00824723_m1
**U6 snRNA (housekeeper)**	Human	001973
**Cel-miR-39 (spike-in control)**	C. Elegans	Cel-miR-39-3p
**VEGF-A (target)**	Swine	Ss0339390_m1
**miR-132 (target)**	Human/Swine	Hsa-miR-132-3p
**miR-210 (target)**	Human/Swine	Hsa-miR-210-3p
**CD31 (target)**	Swine	Ss03392600_u1
**CD45 (target)**	Swine	Ss03376444_u1
**CD11b (target)**	Swine	Ss03374590_m1
**Tbx-18 (target)**	Human	Hs01385457_m1

Total RNA was obtained from cultured APCs (miRNeasy mini kit, Cat n#: 217004, Qiagen) and reverse-transcribed using a High Capacity RNA-to-cDNA Kit (Cat n#: 4387406, Life Technologies), and TaqMan MicroRNA Reverse Transcription Kit (Cat n#: 4366596, Applied biosystem). The reverse transcription–PCR was performed using the first-strand cDNA with TaqMan Fast Universal PCR Master Mix (Cat n#: 4324018, Life Technologies) and on a Quant Studio 6 Flex Real-Time PCR system (Applied Biosystems) for the genes specified above (Table).

Figure S1. Genetic analysis of expanded hAPCs.

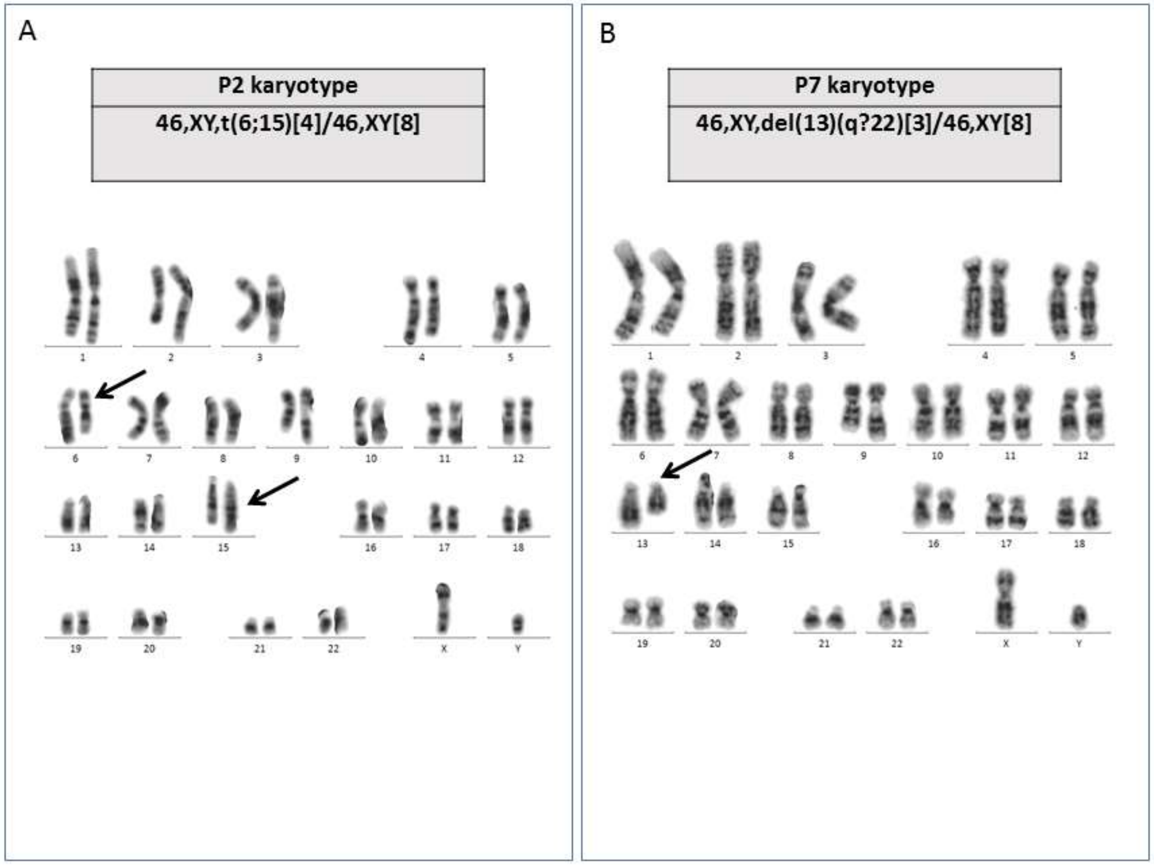

Chromosome analysis was successful in three out of five hAPC lines. Two showed a normal karyotype, while the third showed a balanced rearrangement at P2 (**A**) and an unrelated unbalanced rearrangement at P7 (**B**). *Abbreviations*: hAPC = human Adventitial Progenitor Cells.

Figure S2. Mesenchymal differentiation of sAPCs.

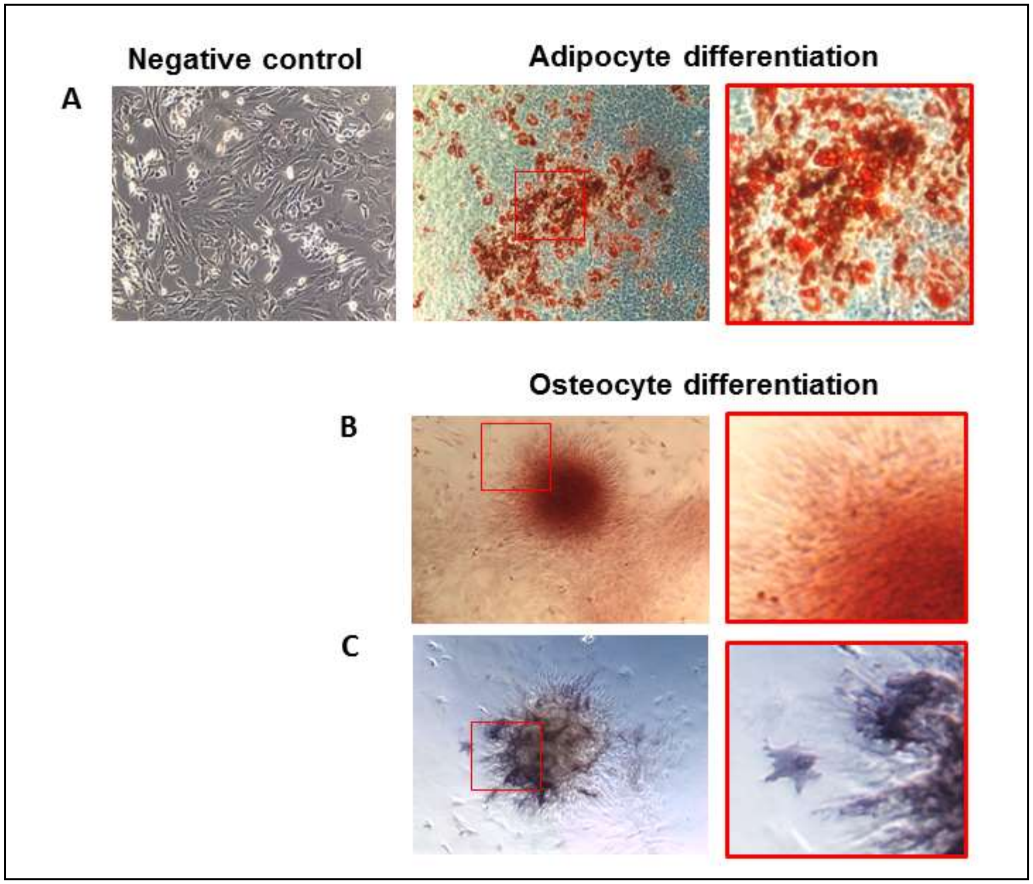

Representative images showing the differentiation capacities of sAPCs towards mesodermal lineages. Lipid droplets were identified by Oil Red staining (red) (**A**) and calcium deposits by Alizarin red (**B**) and ALP staining (**C**) in two sAPC lines. Magnification 100x. Inserts show details of the positive staining. In **A**, sAPCs maintained under normal culture medium are also shown (Negative control). *Abbreviations*: ALP = Alkaline Phosphatase; sAPC = swine Adventitial Progenitor Cells.

Figure S3. Cytotoxic activity of sAPCs and swine splenocytes in suspension.

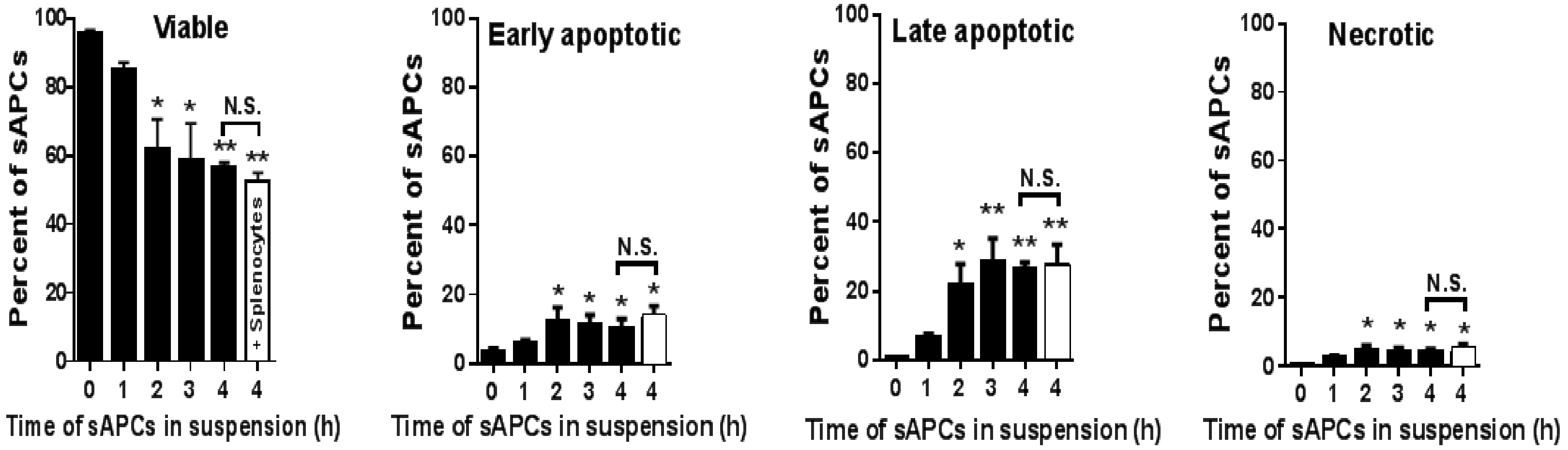

To investigate cell-mediated toxicity, sAPCs (target cells) were incubated alone or with activated swine splenocytes (effector cells). Bar graph showing the percentage of viable (Annexin V^-/P.I.$^-$), early apoptotic (Annexin V^+/P.I.$^-$), late apoptotic (Annexin V^+/P.I.$^+$), and necrotic (Annexin V^-/P.I.$^+$) sAPCs. There was a spontaneous, time-dependent activation of cytotoxicity when sAPCs were maintained alone in suspension for more than 1h. However, incubation of sAPCs with swine splenocytes for 4h in suspension did not further increase the cytotoxic response. Data are expressed as mean±SEM; N=3 biological replicates. *$P<0.05$ and **$P<0.01$ vs. time 0. *Abbreviations*: P.I. = Propidium Iodide; sAPC = swine Adventitial Progenitor Cells.

Supplemental Video Legends

Videos show representative CMR imaging of the hearts at 5 and 45 days after myocardial infarction induction.

Videos S1-2: Vehicle-injected swine (ID# 2644) with CMR imaging captured at 5 day (**1**) or 45 day post-MI (**2**). Format: Windows Media Video file (WMV).

Videos S3-4: hAPC-injected swine (ID#2647) with CMR imaging captured at 5 day (**3**) or 45 day post-MI (**4**). Format: Windows Media Video file (WMV).

Videos S5-6: Vehicle-injected swine (ID#4146) with CMR imaging captured at 5 day (**5**) or 45 day post-MI (**6**). Format: Windows Media Video file (WMV).

Videos S7-8: sAPC-injected swine (ID#4126) with CMR imaging captured at 5 day (**7**) or 45 day post-MI (**8**). Format: Windows Media Video file (WMV).

Supplemental References:

1. Fernandez-Jimenez R, Garcia-Prieto J, Sanchez-Gonzalez J, Aguero J, Lopez-Martin GJ, Galan-Arriola C, Molina-Iracheta A, Doohan R, Fuster V and Ibanez B. Pathophysiology Underlying the Bimodal Edema Phenomenon After Myocardial Ischemia/Reperfusion. *J Am Coll Cardiol*. 2015;66:816-28.
2. Fernandez-Jimenez R, Sanchez-Gonzalez J, Aguero J, Garcia-Prieto J, Lopez-Martin GJ, Garcia-Ruiz JM, Molina-Iracheta A, Rossello X, Fernandez-Friera L, Pizarro G, Garcia-Alvarez A, Dall'Armellina E, Macaya C, Choudhury RP, Fuster V and Ibanez B. Myocardial edema after ischemia/reperfusion is not stable and follows a bimodal pattern: imaging and histological tissue characterization. *J Am Coll Cardiol*. 2015;65:315-23.
3. Garcia-Ruiz JM, Fernandez-Jimenez R, Garcia-Alvarez A, Pizarro G, Galan-Arriola C, Fernandez-Friera L, Mateos A, Nuno-Ayala M, Aguero J, Sanchez-Gonzalez J, Garcia-Prieto J, Lopez-Melgar B, Martinez-Tenorio P, Lopez-Martin GJ, Macias A, Perez-Asenjo B, Cabrera JA, Fernandez-Ortiz A, Fuster V and Ibanez B. Impact of the Timing of Metoprolol Administration During STEMI on Infarct Size and Ventricular Function. *J Am Coll Cardiol*. 2016;67:2093-104.
4. Sanchez-Gonzalez J, Fernandez-Jimenez R, Nothnagel ND, Lopez-Martin G, Fuster V and Ibanez B. Optimization of dual-saturation single bolus acquisition for quantitative cardiac perfusion and myocardial blood flow maps. *J Cardiovasc Magn Reson*. 2015;17:21.
5. Kelley KW, Curtis SE, Marzan GT, Karara HM and Anderson CR. Body surface area of female swine. *Journal of animal science*. 1973;36:927-30.
6. Robbers LF, Eerenberg ES, Teunissen PF, Jansen MF, Hollander MR, Horrevoets AJ, Knaapen P, Nijveldt R, Heymans MW, Levi MM, van Rossum AC, Niessen HW, Marcu CB, Beek AM and van Royen N. Magnetic resonance imaging-defined areas of microvascular obstruction after acute myocardial infarction represent microvascular destruction and haemorrhage. *Eur Heart J*. 2013;34:2346-53.
7. Valle-Caballero MJ, Fernandez-Jimenez R, Diaz-Munoz R, Mateos A, Rodriguez-Alvarez M, Iglesias-Vazquez JA, Saborido C, Navarro C, Dominguez ML, Gorjon L, Fontoira JC, Fuster V, Garcia-Rubira JC and Ibanez B. QRS distortion in pre-reperfusion electrocardiogram is a bedside predictor of large myocardium at risk and infarct size (a METOCARD-CNIC trial substudy). *Int J Cardiol*. 2016;202:666-73.
8. Fernandez-Jimenez R, Silva J, Martinez-Martinez S, Lopez-Maderuelo MD, Nuno-Ayala M, Garcia-Ruiz JM, Garcia-Alvarez A, Fernandez-Friera L, Pizarro TG, Garcia-Prieto J, Sanz-Rosa D, Lopez-Martin G, Fernandez-Ortiz A, Macaya C, Fuster V, Redondo JM and Ibanez B. Impact of left ventricular hypertrophy on troponin release during acute myocardial infarction: new insights from a comprehensive translational study. *J Am Heart Assoc*. 2015;4:e001218.
9. Ayaon-Albarran A, Fernandez-Jimenez R, Silva-Guisasola J, Aguero J, Sanchez-Gonzalez J, Galan-Arriola C, Reguillo-Lacruz F, Maroto Castellanos LC and Ibanez B. Systolic flow displacement using 3D magnetic resonance imaging in an experimental model of ascending aorta aneurysm: impact of rheological factors. *European journal of cardio-thoracic surgery : official journal of the European Association for Cardio-thoracic Surgery*. 2016;50:685-692.
